# Integrated algal and oil palm biorefinery as a model system for bioenergy co-generation with bioproducts and biopharmaceuticals

**DOI:** 10.1186/s40643-021-00396-0

**Published:** 2021-05-20

**Authors:** Mohd Azmuddin Abdullah, Hanaa Ali Hussein

**Affiliations:** 1 Institute of Marine Biotechnology, Universiti Malaysia Terengganu, 21030 Kuala Nerus, Terengganu Malaysia; 2 College of Dentistry, University of Basrah, Basrah, Iraq

**Keywords:** Integrated biorefinery, Algae, Palm oil milling, Bioresource utilization, Bioenergy co-generation, Bioproducts

## Abstract

**Background:**

There has been a greater call for greener and eco-friendly processes and bioproducts to meet the 2030’s core agenda on 17 global sustainable development goals. The challenge lies in incorporating systems thinking with a comprehensive worldview as a guiding principle to develop the economy, whilst taking cognisance of the need to safeguard the environment, and to embrace the socio-cultural diversity dimension as an equal component. Any discussion on climate change, destruction of eco-system and habitat for wildlife, poverty and starvation, and the spread of infectious diseases, must be addressed together with the emphasis on the development of cleaner energy, air and water, better management of resources and biodiversity, improved agro-practices for food production and distribution, and affordable health care, as the outcomes and key performance indicators to be evaluated. Strict regulation, monitoring and enforcement to minimize emission, pollution and wastage must also be put in place.

**Conclusion:**

This review article focuses on the research and development efforts to achieve sustainable bioenergy production, environmental remediation, and transformation of agro-materials into value-added bioproducts through the integrated algal and oil palm biorefinery. Recent development in microalgal research with nanotechnology as anti-cancer and antimicrobial agents and for biopharmaceutical applications are discussed. The life-cycle analysis in the context of palm oil mill processes is evaluated. The way forward from this integrated biorefinery concept is to strive for inclusive development strategies, and to address the immediate and pressing problems facing the Planet and the People, whilst still reaping the Profit.

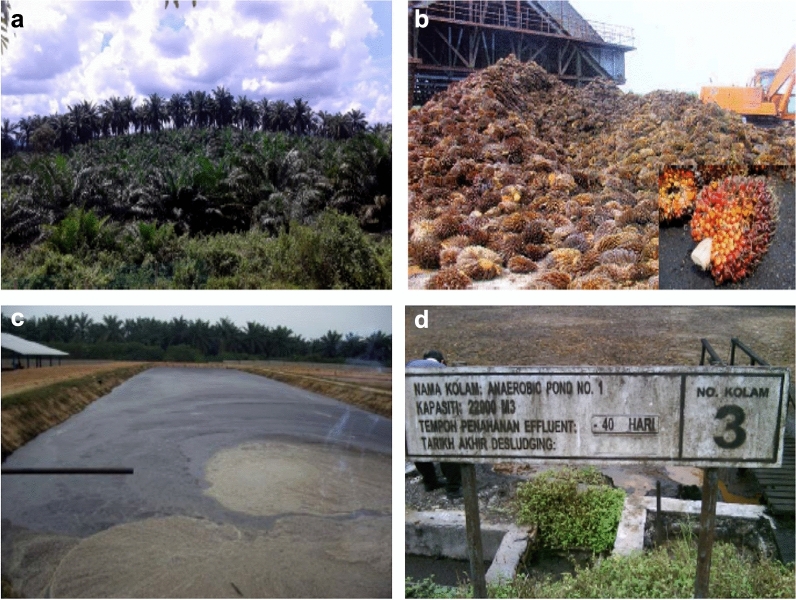

**Supplementary Information:**

The online version contains supplementary material available at 10.1186/s40643-021-00396-0.

## Introduction

Global carbon dioxide (CO_2_) emissions from the fossil fuels have increased 15 times between 1900 and 2008 (Boden et al. 2010). This has prompted greater efforts to develop green technology and eco-friendly materials, to reduce over-dependence on fossil-based fuels and products, and green-house gas (GHG) emissions. Biorefineries aim to achieve efficient and sustainable utilization of biomass resources for the generation of bioenergy and bioproducts (Budzianowski 2017). The biomass conversion processes and equipments are integrated to produce energy, fuels, power and heat, and marketable organic feed, food, chemicals and materials (IEA 2014; NREL 2015). The biorefineries may replace the power plants, or linked to the existing biofuel plants for new biofuels generation, or by re-equipping the existing biofuels with the new bioenergy facilities, or by setting up entirely new facilities, incorporating the processing of the bioresources (Budzianowski 2017; Laosiripojana et al. 2018). The basic principle is to reduce the total raw materials and the consumption of energy per production unit, with the materials being considered within the plant and energy recovery (Chemmangattuvalappil and Ng 2013). As the platform for sustainable production route, the feedstock must be inexpensive and the processes must enable the extraction of bio-energy and bio-products at the highest and maximum possible range (Rathore et al. 2016).

**Fig. 1:**
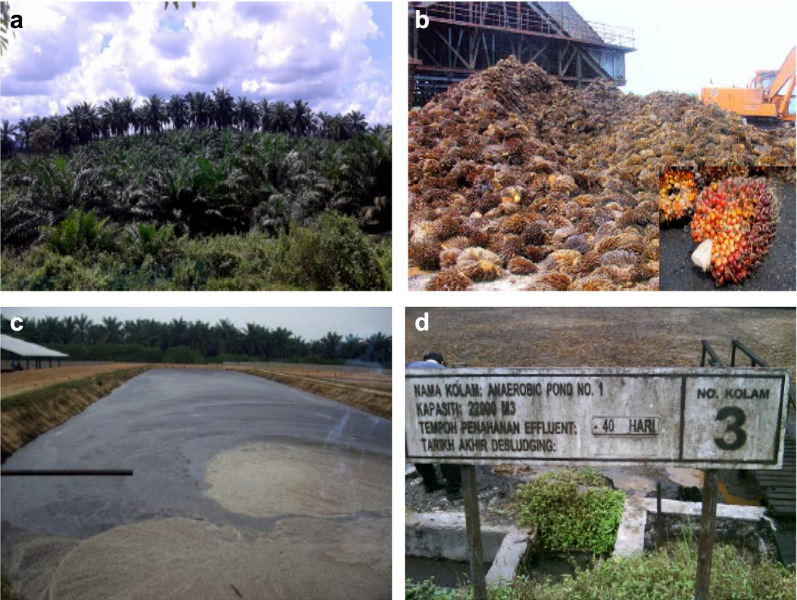
Biomass and effluent generated by the palm oil industry: **a** oil palm plantation, **b** FFBs, **c** open pond, **d** anaerobic treatment pond

Algae are resilience, versatile, and could withstand a variety of weathers and conditions, with less interference in the food supply chain as compared to the conventional oil seed crops (Brennan and Owende 2010; Clarens et al. 2010). Integrated algal biorefinery and palm oil milling (POM) have big potentials to meet the agenda of global sustainable development goals (SDGs) particularly in meeting the demand for affordable and clean energy, developing cities, and surrounding sustainable communities, and practising responsible consumption and production (Abdullah et al. 2016a, b, 2017a; b; Charmondusit et al. 2016; Gheewala et al. 2013). It is crucial to strive for optimal use of locally useful resources while reducing the costs and environmental effects (Pauli 2010). With the combined total of 64.2 million metric tons (MT), Indonesia (56.5%) and Malaysia (27.9%) are the world's largest producers of palm oil (Index Mundi 2019). The palm oil milling processes could generate large amount of biomass residues and effluents (Fig. 1). The fresh fruit bunch (FFB) palm oil is isolated through dry or wet milling methods. The wet method is mostly used which generates high amount of palm oil mill effluent (POME). This has created an environmental load because of the huge discharge of wastewater during the milling process. It is estimated that 5–7 tonnes of fresh water are needed to process 1 tonne of FFB, of which 50–79% ends up in POME (Ohimain and Izah 2017). The residues generated, depending on the typical biomass of the fresh fruit bunches (FFBs) extraction rate (on wet basis percentage) are empty fruit bunches (EFBs) (22), mesocarp-fibres (MF) (13.5), and palm kernel-shells (PKS) (5.5) and POME (67) (Loh 2016). The development of an integrated refinery is essential for sustainable conversion of EFB, MF, PKS, and POME into high value products (Theo et al. 2017).These provide great resources for conversion into value-added products. Technologies such as composting, pelletizations, agglomeration, compression, pyrolysis, co-generation, enzymatic/acid/alkali digestion or autoclave/heat/steam treatment can be applied for the conversion of the residual biomass (Chiew and Shimada 2013; Nazir et al. 2013; Chang 2014). The shells and the fibres can be utilized for steam and electricity generation in the mill (Nasrin et al. 2008). The EFBs are re-used as fertilizers by mulching in the plantation or disposed of in the landfill or burnt to produce potash (Chavalparit et al. 2006). The palm kernel cakes are composted, or for animal feeds (Singh et al. 2010). The POME is normally treated in the ponding system in sequence, consisting of the anaerobic, facultative, and aerobic ponds, which all require low investment costs (Abdullah and Ahmad 2016). A new agro-production model based on the co-cultivation of microalgal biomass within the POM setting will be attractive for regenerating biofuels (e.g. ethanol, methanol, bio-oil, and biodiesel) (Sawaengsak et al. 2014; Garcia-nunez et al. 2016), environmental remediation with biogas production, briquettes, biomass fuel pellet, and dried long fibres (Abdullah and Ahmad 2016; Abdullah et al. 2016a; Theo et al. 2017), conversion into value-added biomaterials (Abdullah et al. 2016b; 2017b), and as a route to the production of high-value biocompounds and bioproducts (Abdullah et al. 2016a, 2017a).

There has been an increasing interest in the biologically active metabolites from natural sources for their beneficial effects on health (Herrero et al. 2013). This has led to the search for novel active pharmaceutical compounds and the development of novel drugs to treat various human diseases (Newman and Cragg 2012). Several clinically usable and commercially available drugs from natural products have shown potency as anti-tumour, antiallergy and anti-infection activity and capable of treating analgesia and cognitive diseases (Bhattacharjee 2016). Microalgae have the potential to improve health and cure many diseases (De Morais et al. 2015). Being one of the ancient living organisms on Earth, microalgae can grow in diverse habitats such as the desert and the sea (Stengel et al. 2011). This varied habitat leads to many biologically effective therapeutic metabolites as a response to multitude of stressors that can be extracted from the biomass or produced extracellularly (Bhagavathy et al. 2011). Microalgae is ideal as an alternative system to molecular pharming as they do not contain pathogens for human and are generally regarded as safe (GRAS) organisms. Microalgae can grow in axenic conditions in controlled bioreactors that could facilitate the production of biopharmaceuticals, and achieve the homogeneity of proteins, antibiotics and phytochemicals (Specht et al. 2010; Abdullah et al. 2016a, 2017a). The bioactive molecules extracted from microalgae have exhibited the ability to reduce or prevent diseases (Volk 2008). These compounds may be primary metabolites such as fatty acids, proteins, pigments and vitamins, or secondary metabolites such as the terpenoids or phenolics compounds (De Morais et al. 2015). Others include lipids, polysaccharides, antioxidants, carotenoids, chlorophylls, sterols, enzymes, flavonoids or small molecules of pharmaceutical and nutritional importance (Priyadarshani and Rath 2012). Of great interest is in the co-application of natural products with nanotechnology to improve the therapeutic efficacy with reduced side-effects; or enhance the bioactivities/antimicrobial activities of existing drugs/antibiotics (Hussein et al. 2020a, b). Optimal engineering involving microalgal biofuel production and the recovery of co-products, with the environmental and molecular factors, could trigger constitutive product accumulation including the lipids, carbohydrates and high-value bioproducts.

Algal cultivation for food and animal feed could meet the agenda of global food security. The global aquaculture production of farmed aquatic animals between 2001 and 2018 grew at 5.3% annually, on average, and the total fish production is projected to increase from 179 million tonnes in 2018, to 204 million tonnes in 2030. The global production of marine macroalgae has expanded from 10.6 million tonnes in 2000, to 32.4 million tonnes in 2018 (FAO 2020). However, for large-scale production of algal biochemicals and biofuels, the development of low volume, high-value chemical products is needed. Currently, the microalgal manufacturing infrastructure has been focusing on the extraction of high-value products (IEA Bioenergy 2012). The annual production of microalgae is 5000 tonnes dry matter per year with the global turnover of around US$1.25 × 10^9^ per year, while the macroalgal production is 7.5 × 10^6^ tonnes dry matter per year with 4.8-fold higher global market than the microalgae (Pulz and Gross 2004). To achieve economic viability, the business model for fast adoption of algal technology is to use the entire biomass, with improved technology for cultivation and to focus the production on high lipid, protein and carbohydrate contents, with other valuable compounds such as fatty acids, pigments, antioxidants, vitamins, anti-microbial, toxins, sterols, nutraceuticals and pharmaceuticals (IEA Bioenergy 2012; Ubando et al. 2020). The production of bulk chemicals and energy from microalgae must be implemented at larger scale (increase of 3 orders of magnitude) and at much lower costs (a reduction by a factor of 10) (IEA Bioenergy 2012). Biorefinery is the way forward to green the economy and to achieve sustainability by achieving the balance between the environmental, societal and economic governance (Giraldo et al. 2020). The integrated biorefinery approach producing biofuels and value-added products has higher potential of gaining economic profitability (Rajesh Banu et al. 2020). Sequential extractions of products before anaerobic digestion, for example, could improve the methane yields and make the overall process more viable (Mhatre et al. 2019). Fractionation of algal biomass into major constituents—lipids, carbohydrates and proteins, and separation and purification into other value-added bioproducts in a biorefinery would optimize the economics, whilst minimizing the energy, water, nutrients, land use and environmental footprint (Laurens et al. 2017; Abdullah and Hussein 2020). The process integration for the co-production of diverse products must therefore be evaluated such that newer products, applications and markets can be developed (Gajaria et al. 2017).

The aim of integrated algal and oil palm biorefinery is to achieve effective utilization of biomass resources, with efficient organizational and administrative procedures, goods, services, equipments and logistics, leading to increased economic value of multiple products including biofuels, biochemicals, biopharmaceuticals, and foodstuffs. These eventually could increase the utilization of renewable resources, whilst reducing the use of non-renewable resources, and expand the storage, treatment and disposal of wastes (Giraldo et al. 2020). A cascading biorefinery can be developed to enhance the algal economic value chain through the valorization of proteins, lipids, starch/polysaccharides, vitamins and minerals for food, animal feed, biofuels and also multiple bioindustries (van Hal et al. 2014; Masarin et al. 2016; Bleakley and Hayes 2017; Gajaria et al. 2017). Biomass wastes and waste water generation will push the boundaries of conversion into value-added products, but the promotion of circular economy will be very much influenced by the socio-economic factors which are the major barriers in adopting biorefineries (Ubando et al. 2020). Despite the vast potential, the opportunities to valourize the wastes and waste water and extract economic values from palm oil mill and oil palm plantation have not been fully developed. Large amount of agro-industrial and industrial wastes can be recycled and reused as a nutrient supply for algal cultivation, and converted into bioenergy. The oil palm biomass can be pre-treated, and the bulk chemicals extracted and utilized for diverse applications. The wide spectrum of microalgal metabolites including alkaloids, polyphenols, flavonoids, and carotenoids, exhibiting a broad range of biological activities, must be exploited and refined for pharmaceutical industries. For these, the specific conditions must be developed and optimized such that algal utilization is fit for human consumption as food, or as feed for aquaculture (Abdullah et al. 2017a, b; Shah and Abdullah 2017).

This paper reviews the clean energy initiatives in oil and gas industries, the biorefinery concept based on the cultivation of algae and palm oil milling for the production of bioenergy, and the value-added bioproducts co-generation, with the applications of bio/nanotechnology platform.

### Clean energy initiatives in oil and gas industries

The initiatives made by the oil and gas industries in energy sector must be understood to better appreciate the need for adaptability and a smooth transition into the development of an integrated biorefinery. Efficient utilization of energy, higher supply and availability of clean fuels, elimination of pollutant emission and reduction of GHGs are attainable by utilizing Hydrogen (H_2_) and Syngas (a mixture of H_2_ and CO). Hydrocarbon fuels need a hydrocarbon fuel processing system such as catalytic steam reforming (CSR), partial oxidation and autothermal reforming (ATR), oxidative steam reforming, water–gas shift or preferential oxidation of natural gas, ethanol biogas, or biomass, for conversion into H_2_ or syngas fuel (Subramani et al. 2010; Kaiwen et al. 2018). The electrical power generation based on the syngas can be implemented through the conventional and advanced technologies involving natural gas combined cycle (NGCC) and integrated gasification combined cycle (IGCC) plants utilizing both gas and steam turbine, internal combustion engines (ICEs) for transportation and distributed power generation, solid oxide fuel cells (SOFCs) (Williams et al. 2006), and molten carbonate fuel cells (MCFCs) and the hybrid plants with turbines and fuel cells. The two most common methods to produce hydrogen are steam reforming of a natural gas and gasification of coal. The CSR technique produces H_2_-rich gas, from a hydrocarbon, or alcohol fuels and water, over a base metal, or noble metal-supported catalysts (Subramani et al. 2010). The gasification of coal is economically feasible but need to address the concerns on the environmental impacts and high CO_2_ emission. Natural gas has been the main feedstock for H_2_ production and the method is well-established, requiring low capital cost at $2.48–3.17/kg H_2_. The transportation costs of natural gas are also lower than hydrogen (Bartels et al. 2010; Kaiwen et al. 2018). For environmentally effective route, the steam reforming of biogas to produce hydrogen and methanol is viable, which may reach the value of $0.27/kWh for H_2_ (Braga et al. 2013), and an estimated $1.75/gal for methanol (Hernandez and Martin 2016). Methanol, in turn, can be catalytically converted into other transportation fuels such as gasoline, dimethyl ether or biodiesel. Ethanol steam reforming is also attractive but the costs for H_2_ production can be influenced by the plant capacities, the catalysts and the ethanol feedstock. Low plant capacity can result in higher H_2_ selling price, and increasing the plant capacity by 100-fold from 1500 kg/day can reduce the selling price to $2.69/kg H_2_, but the capital cost may be increased. To compensate, the cost of catalysts can be made more acceptable and the total cost of hydrogen production reduced, if ethanol is sourced from the fermentation of biomass (Song and Ozkan 2010).

Methane produced from natural gas or biogas, has high calorific value, and is suitable for large-scale steam reforming route (Kaiwen et al. 2018). The important factors in the steam reforming of methane for H_2_ production are the reaction pressure, temperature, the selectivity of H_2_, and the H_2_/CO ratio (Farshchi Tabrizi et al. 2015); and the length of the reactor (Imran Aman et al. 2017). The combined steam reforming and dry reforming of methane exhibit lower char footprint, but requiring additional equipments, which incur higher total direction cost, as compared to the steam reforming alone (Gangadharan et al. 2012). The sorption enhanced steam methane reforming with in situ CO_2_ capture could be used for H_2_ production from natural gas, with high purity and GHG sequestration. The Ni–mayenite support system developed for the process exhibits good performance, while the combined sorbent catalyst material based on Ni–CaO–mayenite is found to have the Ni catalytic activity lowered by the presence of CaO loading (Di Giuliano et al. 2017). The sorption enhanced steam reforming of methane in a network of fixed beds, with the integration of a SOFC, has been evaluated for power generation. The cost of H_2_ production with CO_2_ capture is levelled at $1.89/kg, while the cost of CO_2_ avoided is USD$35.3/tonne CO_2_. These are 1.5 and 1.7-fold, respectively, lower than the cost of reforming without the sorption. The integration with the SOFC also levels the cost of electricity at $0.092/kWh with the cost of CO_2_ avoided at $43.5/tonne CO_2_. These are comparable and 2.1-fold lower, respectively, than the natural gas-fired power plant with the carbon capture. The economic analyses further suggest the possibility of attaining higher revenue and the feasibility of implementing the sorption enhanced steam methane reforming, with the network of fixed beds integrated to the SOFC, for hydrogen production and CO_2_ capture, as an alternative to the natural gas-fired power plant (Diglio et al. 2017). The catalysts used in the steam reforming of methane for H_2_ production are mostly Ni-based, attributable to its high reactivity as compared to the natural catalyst. The important natural catalysts such as dolomite and olivine, have tremendous potential for application in the production of H_2_-rich gas and CO_2_ capture, but the efficiency may be lower than the Ni-based catalyst (Kaiwen et al. 2018).

The major drawback of steam reforming method is the high amount of unconverted hydrocarbon (tar) in the produced gas which necessitates further processing (Kaiwen et al. 2018), and the carbon deposition and catalyst deactivation. Further improvement in the catalyst development and reactor design are therefore required (Subramani et al. 2010). The hot and warm gas cleanup must be put in place for particle removal, carbonyl sulphide (COS) hydrolysis, acid gas cooling, sulphur and mercury removal and recovery, and CO_2_ sequestration. The char and fly ash can be removed by cyclone filters, ceramic or metal candle filters, or wet scrubbing. Sulphur compounds or hydrogen sulphide (H_2_S) are poisonous to the catalysts, the fuel cell stacks, or the separation membrane for H_2_ purification. Ultradeep desulfurization of hydrocarbon fuels is required to reduce the sulphur contents to lower than 15 ppm for diesel, or 30 ppm for gasoline. The technologies for desulfurization include catalytic hydrodesulfurization (HDS), new design and properties of the catalyst, reactor, and the process, and multidesulfurization technologies such as adsorptive (ADS), oxidative (ODS), extractive (EDS) and biodesulfurization (BDS) (Nishioka et al. 1985; Tuan et al. 1994; Dicks 1996; Song and Ma 2010). The future lies in high efficiency and low emission technologies based on the NGCC, IGCC and hybrid fuel cell power plants (Song 2010; Wei et al. 2010). The syngas production and purification technologies are critical to gas-to-liquid (GTL) and biomass-to-liquid (BTL) conversion systems. The syngas from solid and gaseous fuels can then be the feedstocks to synthesize liquid hydrocarbon fuels, methanol, dimethyl ether and ethanol for transportation vehicles (Song 2010). These should be the major considerations and become the basis for the transition into an integrated biorefinery.

### Integrated algal and oil palm biorefinery

#### Bioenergy co-generation

The clean development mechanism for energy sector may involve the renewable energy generation based on hydropower or biomass; the replacement of coal with natural gas or biomass; the improvement of energy efficiency by utilizing more efficient motors or equipments with co-generation of heat and power; and the capture of biogas and methane to generate energy (Mekhilef et al. 2011). The oil palm industries have big potentials to be the next generation renewable energy providers and feedstocks for biorefineries. The oil palm tree has a higher efficiency of oil production at 4000 kg/ha as compared to the sunflower, soybeans, peanuts, cotton, and rapeseed. The oil palm tree also has a relatively long economic lifespan of 25 to 30 years, with a reliable supply of oil production (Kurnia et al. 2016). The technologies to generate electrical energy from biomass sources may involve direct combustion of biomass such as EFB or mesocarp fibres; gasification of lignocellulosic biomass into hydrogen gas; pyrolysis; briquetting of EFB or palm kernel expeller (PKE) into solid fuels; and anaerobic digestion (Mekhilef et al. 2011). The EFBs can be converted into bioethanol by hydrolysis, fermentation, and distillation, or to bio-oil by rapid pyrolysis and gasification (Abdullah et al. 2011; Ishola et al. 2014).

### Biodiesel

Palm oil exhibits superior content (on the weight basis) of palmitic acid (C16:0) (45%), as compared to other edible oils from soybean, sunflower, rapeseed, corn and coconut, but with comparable content of stearic acid (C18:0) (4%), oleic (C18:1) (39%), and lower linoleic acid (C18:2) (11%). In comparison, *Chlorella* sp. shows 24–36% palmitic, 1–2% stearic, 13–17% oleic, and 33–41% linoleic acid (Zahan and Kano 2018). These are the fatty acids constituents normally associated with the transformation of oil into a high quality biodiesel. The refined crude palm oil (CPO) is transformed into methyl esters and used directly or blended with petroleum diesel. The transesterification method has also been developed for refined, bleached and deodorized (RBD) palm olein with methanol, in the presence of potassium hydroxide (KOH) catalyst to achieve 62.5% yield, which is lower than that achievable with the palm oil (95.3%) (Kareem 2017) and palm kernel oil (94.6%) (Akhabue and Ogogo 2018). The derived biodiesel however shows comparable density of 884 kg/m^3^, and kinematic viscosity of 4.56 mm^2^/s at 40 °C, to the palm oil and palm kernel oil, although slightly higher than diesel at 850 kg/m^3^ (Ishola et al. 2020). The cetane number attained at 48.91 is comparable to the ASTM standard, though slightly lower than diesel at 50. The flash point of RBD palm olein biodiesel at 208 °C, though in the middle between palm oil (270 °C) and palm kernel oil (162 °C), is far higher than diesel (85 °C), and the minimum of ASTM standard (52 °C) (Hariram et al. 2018; Ishola et al. 2020). The biodiesel from non-edible oil source (*Moringa oleifera*) has been compared with palm biodiesel and diesel fuel. The palm and *M. oleifera* biodiesel and the blends, although meet the ASTM D6751 and EN14214, exhibit lower brake powers and higher brake specific fuel consumption at the 5% blends. However, the biodiesel at 5 and 10% blends of both oils have resulted in reduced average emissions of carbon monoxide and hydrocarbons, but with increased nitric oxide CO_2_ emissions, as compared to the diesel fuel (Mofijur et al 2014).

### Bioethanol

EFB contains approximately 73.6% (w/w) holocellulose and the degradation of cellulose produces glucose as the main product which can be used for the conversion into bioethanol (Laosiripojana et al. 2018). Bioethanol production from lignocellulosic biomass requires pretreatment or delignification step to release cellulose and hemicellulose. The hydrolysis of cellulose and hemicellulose produces sugars which will be fermented to produce ethanol (Sukhang et al. 2020). The pretreatment of EFB with 1% (v/v) dilute H_2_SO_4_ at 125 °C for 90 min, followed by 1% (w/v) NaOH at 100 °C for 60 min, removes more than 90% of hemicellulose and 50% of lignin. The delignified EFB at 5% (w/v) is enzymatically hydrolysed for 72 h to attain about 485 mg/g glucose. The addition of Triton-X enhances the saccharification by 31.3%. The fermentation of the derived sugar by *Saccharomyces cerevisiae* produces 12 g/L of bioethanol with 89.1% theoretical yield within 24 h (Nurul Adela et al. 2014). The SSF of the oil palm frond (OPF) with *Saccharomyces cerevisiae* has been carried out for bioethanol production. The pretreatment of the OPF at 20% (w/v) by pre-soaking in different acid or alkali, results in the cellulose yield of 37% in 2% H_2_SO_4_, 42% in NaOH, and 49% in 2% NaOH in H_2_O_2_. The simultaneous saccharification by cellulase produces 45.72, 55.73 and 56.94 g/L sugar yield, respectively, and the bioethanol production of 14.5, 15.0 and 17.2 g/L, respectively. With the total solids recovery of 82.11%, containing 49% cellulose, and 37.6% enzyme digestibility, the 2% NaOH in H_2_O_2_ is suggested to be the best pretreatment method (Kumneadklang et al. 2015). The acid–alkali pretreatment of EFB using a dilute acid of 0.2 M sulphuric acid concentration (12.5% (w/v)), 121 °C for 20 min, has resulted in 72.1% cellulose, 3.24% hemicellulose, and 17.6% lignin. The optimal conditions for simultaneous saccharification and fermentation (SSF) by *Klyveromyces marxinus* are at 12.24% substrate, pH 4.5, 2.04% (v/v) yeast, and 36.94 °C, to produce 0.281 g/g bioethanol. With separate hydrolysis and fermentation (SHF) at optimal conditions, 0.584 g/g reducing sugars and 0.258 g/g bioethanol, are produced. The acid–alkali pretreatment therefore could achieve high delignification of the lignocellulosic biomass, and increase the cellulose yield, with the SSF achieving faster processing time and higher bioethanol production (Sukhang et al. 2020). The production of bioethanol as a part of palm oil processing could result in positive environmental impact in general, but may reduce the net energy ratio (NER) by 27.5%, the net carbon emission ratio (NCER) by 66.6%, and the carbon emission savings (CES) by 21.9%. This suggests that a higher amount of energy input and GHG emissions is needed for bioethanol production, than the amount of energy it will provide and the GHG from the fossil fuels it will displace (Lim et al. 2011).

### Biohythane

The biogas produced from the biological decomposition of biomass or agricultural residues, in anaerobic conditions, can be an economically and reliable source of renewable energy. The microbial bioreactions to generate biogas include acidogenesis, hydrolysis, methanogenesis, and acetogenesis, of biomass or effluents. The biogas constituents from anaerobic digestion typically are methane (60%), carbon dioxide (35%), hydrogen sulfide (3%), hydrogen (1%) and other gases (Wooster 2009). For biohydrogen production, the dark fermentation of POME, has been evaluated in the two-stage thermophilic (55 °C) and mesophilic (37 °C) anaerobic sequencing batch reactor (ASBR), with enriched mixed culture. The effluent from the thermophilic reactor contains 7.61 g/L total carbon (TC) and 22.87 g/L total suspended solids (TSS), which are then fed into the second mesophilic reactor. The thermophilic stage records the optimum H_2_ yield of 2.99 mol H_2_/mol sugar, and H_2_ production rate of 8.54 mmol H_2_/L.h, while the mesophilic stage registers 1.19 mol H_2_/mol sugar and 1.47 mmol H_2_/L.h. The overall improvement is an increase from 8.54 to 10.34 mmol H_2_/L.h (Maarof et al. 2019). The two-stage dark fermentation and microbial electrolysis under thermophilic condition has also resulted in maximum POME conversion into biohydrogen with the maximal yield of 0.236 L H_2_/g carbon oxygen demand (COD) and 7.81 L H_2_/L POME.day. The yield is 3 times higher than the dark fermentation alone, and the dark fermentation effluent is rich in acetate and butyrate (Khongkliang et al. 2019).

The two-stage thermophilic fermentation and mesophilic methanogenic process utilizing POME have exhibited the H_2_ potential of 170–200 L H_2_/kg COD and the CH_4_ potential of 210–292 L CH_4_/kg COD. The two-stage process in the continuous mode with 2 days Hydraulic Retention Time (HRT) for H_2_ reactor attains 210 L H_2_/kg COD, and 15 days HRT for CH_4_ reactor achieves 315 L CH_4_/kg COD, with the total energy yield of 15.34 MJ/kg COD. This is a 34% higher energy yield than the single stage CH_4_ reactor. The total production rate of biogas is 4.4 L /L POME.day, comprising 51% CH_4_, 14% H_2_ and 35% CO_2_ (Mamimin et al. 2015). Utilizing the batch process of two-stage thermophilic fermentation and mesophilic methanogenic, and with 30% POME recirculation, maximum of 4.1 L H_2_/L POME and 16.6 L CH_4_/L POME, are recorded. The continuous mode of operation, with and without recirculation, achieve slightly lower biogas at 3.8 and 2.2 L H_2_/L POME, and 14 and 12.2 L CH_4_/L POME, respectively. The yields of 0.135 L H_2_/g Volatile Solids (VS) and 0.414 L CH_4_/g VS are attained, with the biogas composed of 54.4% CH_4_, 13.3% H_2_, and 32.2% CO_2_ (Thong et al. 2016). The pilot scale of two-stage thermophilic fermentation has been operated at thermophilic condition (55 °C), with organic loading rates (OLR) of 27.5 g COD/L.day and 2 days HRT in the first stage, and OLR of 5.5 g COD/L.day and 10 days HRT in the second stage. The biogas mixture of 52% CH_4_, 11% H_2_, and 37% CO_2_, with the biohythane production rate of 1.93 L gas/L.day, are registered. The recirculation of methane mixed with POME (at the 1:1 ratio) provides control of the pH at 5–6.5 in the first stage. The H_2_/CH_4_ ratio of 0.13–0.18 is suggested to be suitable for use as vehicle fuel (Seengenyoung et al. 2019).

The effluent rich in H_2_ from the decanter cake (DC) and the crude glycerol (CGL) co-digestion have been evaluated in the two-stage thermophilic H_2_ fermentation, and mesophilic methanogenic process for CH_4_ production. The single stage H_2_ production, using 2% (w/v) total solid (TS) of DC and CGL, in 4 days HRT with optimally loaded 1.5% CGL co-digestion, is 0.461 L H_2_/L POME.day and 23 L H_2_/kg TS. The semi-continuous mode of CH_4_ production, utilizing 0.75% CGL effluent, achieves 0.736 L CH_4_/L POME.day and 44 L CH4/kg TS. The total energy recovery is 0.056 kWh/kg TS (Kanchanasuta et al. 2017). The biohythane production utilizing co-digestion of oil palm solid waste residues with POME in two-stage thermophilic fermentation has been developed, resulting in biohythane yield of 26.5–34 m^3^/ton waste, which is 67–114% increase, as compared to the POME digestion alone. The co-digestion of solid wastes with POME enhances the hydrolysis constant (*k*_*h*_) from 0.07–0.113 to 0.12–0.223/day, which is 10 times higher than the single digestion. During hydrogen stage, *Clostridium* sp. predominates, while *Methanosphaera* sp. predominates the methane stage (Mamimin et al. 2019). The EFB has also been investigated as the potential sustainable source of biohydrogen production by electrolysis technique. The dried EFB, cut into small pieces, and FeCl_3_.6H_2_O as an oxidizer at different concentrations, are refluxed at 90–98 °C for 5 h, in the presence of 10% (v/v) HCl in deionized water. The filtered aliquot is further diluted to 1:10 ratio to reduce acidity, and made to undergo electrolysis using the titanium anode and stainless steel cathode, at 15 V direct current. The results suggest that the higher the oxidizer concentration used, the longer the time it takes to produce H_2_ gas using the aliquot with longer storage time. The fresh aliquot however records higher volume of H_2_ gas generated experimentally than that predicted theoretically (Amri et al. 2019).

Different types of microbial species and community have been identified during biohythane production, incorporating different mode of operations and configurations. *Thermoanaerobacterium* sp. has been found the predominant microbial community during the dark fermentation of POME (Maarof et al. 2019; Khongkliang et al. 2019) and during the H_2_ stage in the two-stage process with methanogenic effluent recirculation (Thong et al. 2016), and in the pilot scale of the two-stage thermophilic fermentation (Seengenyoung et al. 2019). The microbial electrolysis cell stage is dominated by *Geobacter* sp. and *Desulfovibrio* sp. (Khongkliang et al. 2019). In the two-stage thermophilic fermentation and mesophilic methanogenic process utilizing POME, the H_2_-producing bacteria *Thermoanaerobacterium thermosaccharolyticum* predominates the H_2_ reactor, and the acetoclastic *Methanoculleus* sp. is the dominant methanogen in the CH_4_ reactor (Mamimin et al. 2015). However, *Methanosarcina* sp. dominates the CH_4_ stage during the pilot scale of two-stage thermophilic fermentation (Seengenyoung et al. 2019). The biohythane production utilizing co-digestion of oil palm solid waste residues with POME in two-stage thermophilic fermentation suggest that *Clostridium* sp. predominates during H_2_ stage, while *Methanosphaera* sp. predominates the CH_4_ stage (Mamimin et al. 2019).

### Microalgal POME treatment and bioenergy co-generation

The National Renewable Energy Laboratory (NREL) is among the pioneer in the research and development of algal biofuels (NREL 2015). As energy producers, algae are characterized by simple cellular structure, and rapid reproduction rate as compared to the terrestrial plants, allowing for multiple cultivation and harvesting period in a year. Algae can grow in saltwater or wastewater, whilst fixing carbon dioxide. The algal lipid contents may be in the range of 30–80% on the dry weight basis, with the oil yield of 10–800 times more than the conventional crops on per area basis (Chisti 2007; Abdullah and Ahmad 2016; Suganya et al. 2016). The microalgal lipids can be converted into biodiesel; carbohydrates (starch and cellulose) for bioethanol; and the residual fats, carbohydrates, and proteins in microalgal tissues can be transformed into biohythane by anaerobic digestion. The chemical, biochemical, and thermochemical conversion processes could produce syngas, butanol, jet fuel, and bio-oil, or for human nutrition, fine chemicals, medicine, cosmetics, and animal feed (Zhu 2015). One of the most effective way to produce algal biodiesel is to integrate with the waste-water treatment involving high technology for waste water remediation and biomethane or biohydrogen generation.

Algal cultures can be divided into photoautotrophic, heterotrophic, mixotrophic, and photoheterotrophic cultivation (Piasecka et al. 2020; Debowski et al. 2020). Autotrophic microalgae use light from photosynthesis to convert CO_2_, water and minerals, to grow and synthesize biocompounds. Heterotrophic microalgae can grow in the dark using organic compounds like carbon (such as sucrose, glucose, fructose, glycerol) as energy sources, while mixotrophic microalgae can use both photosynthesis and organic and inorganic carbon substrates. Heterotrophic cultures exhibit higher growth rates and biomass/lipid productivity than the phototrophic and mixotrophic cultures (Debowski et al. 2020). However, bacterial contamination in heterotrophic mode may affect the biomass and lipid concentration although the extent of nitrogen and phosphorous degradation may be improved (Zhang et al. 2012). The cultivation of microalgae on waste water as a growth medium is more economical as the water and nutrients are readily available, while the high CO_2_ levels in the wastewater promote algal growth which releases O_2_ for the bacterial community in the waste water to further enhance the pollutant degradation rate (Wang et al. 2008; Molazadeh et al. 2019). Algae therefore play the dual role of taking up the nutrients and supplying oxygen to bacteria. The bacteria in turn participate in the breakdown of the organic matter in the waste-water, the same process as used in activated sludge. However, microalgae are able to decrease the COD and Biological Oxygen Demand (BOD) in waste-water to get rid of pathogens, nitrogen, and phosphorus, in a more economical way than the activated sludge (Singh and Dhar 2011). In high-rate algal pond (HRAP) containing photobioreactor and intensified oxidation ponds, microalgae will provide oxygen for the bacteria, while the bacteria convert the minerals such as ammonium into nitrate, which is used as a nutrient for microalgae (Molazadeh et al. 2019). The species suitable for waste-water treatment include *Scendesmus* sp., *Chlamydomonas reinhardtii*, and *Chlorella* sp. Algal species such as *Sargassum, Lamiaria, Ecklonia, Macrocystis, Durvillaea, Ulva*, and *Lessonia*, readily adsorb hazardous heavy metal ions from the environment, through binding factors and proteins. (Abdullah and Ahmad 2016). Integrated processes of algal cultivation and waste-water treatment for biomethane production can decrease the cost associated with the CO_2_ biological mitigation. One of the main challenges in algal application for the waste treatment is to define a method that allows for a proper post-treatment for the biofuels production and other bioproducts (Christenson and Sims 2011).

Algal co-cultivation can be implemented to attain sustainable energy management in the palm oil mill for bioenergy co-generation, with environmental remediation and biochemicals production (Sawaengsak et al. 2014; Abdullah et al. 2015; 2016a; 2017a, b). The bioreactor configurations utilized for anaerobic decomposition of POME include up-flow anaerobic sludge blanket reactor (UASB), expanded granular sludge bed reactor (EGSB), anaerobic baffled bioreactor (ABR), modified anaerobic baffled bioreactor (MABB), up-flow anaerobic sludge fixed-film reactor (UASFF), continuous stirred tank reactor (CSTR), membrane anaerobic system (MAS), ultrasonicated membrane anaerobic system (UMAS), and ultrasonic-assisted membrane anaerobic system (UAMAS) (Abdullah and Ahmad 2016; Ohimain and Izah 2017). The EFB and palm-kernel can be used as the co-substrate to the sludge inoculum and POME to achieve high biogas production rate of 0.0574 m^3^/kg COD.day, and 25.6% methane, at 47.8 °C, in a 500 mL reaction vessel (Saleh et al. 2012). The residual biomass of *Chlorella* from anaerobic solid-state fermentation has been pre-treated with acid, thermal and acid-thermal methods and the hydrolysates produced are used in the dark fermentation for hydrogen production, followed by methanogenesis during anaerobic digestion to produce biomethane. The acid-thermal pretreatment method produces a maximum content of 28.9 mg reducing sugar/g biomass, resulting in the highest hydrogen of 12.5 mL/g Volatile Solid (VS), and biomethane of 81 mL/g VS. The estimated total energy yield is 3.03 kJ/g VS or 4.6% energy recovery, based on the heating value of the residual biomass (Lunprom et al. 2019). Mono-algal co-digestion of *Chlorella* sp. at 0.12 g/mL EFB and POME of 2 mL/mL attains the highest biomethane rate of 5.29 L CH_4_/L POME.day, with high removal of COD (98%), BOD (95%), total nitrogen (TN) (78%) and, total organic carbon (TOC) (78%) after 7 days of anaerobic treatment (Ahmad et al. 2014). The co-cultivation of *N. oculata* at 2 mL/mL POME and EFB of 0.12 g/mL POME achieves 4.61–5.02 L CH_4_/L POME.day, with high removal efficiencies of COD (90–97%), BOD (84–98%), and TOC (65–80%) (Ahmad et al. 2015).

With multi-algal anaerobic co-cultivation of *Nannochloropsis oculata* and *Chlorella* sp., each at 1 mL/mL POME, with EFB of 0.12 g/mL POME (Table 1), the highest biomethane (4.65 L CH_4_/L POME.day) and the specific biogas rate (0.124 m^3^/kg COD.day) with CO_2_ (2.27 L CO_2_/L POME.day) are obtained (Ahmad et al. 2016). Microalgal co-cultivation with POME, EFB as co-substrate, and POME sludge as an inoculum therefore not only improves the biomethane production, but also the POME remediation. The microalgae and EFB co-substrate addition could potentially enhance the buffering capacity of the digester (Abdullah and Ahmad 2016), and complement the role of anaerobic bacteria during the digestion process. POME is rich in organic and inorganic contents, and filtered POME can be developed as an economical alternative media with seawater for microalgal cultivation to promote cell growth, lipid and fatty acids accumulation (Table 2) (Shah et al. 2014a, b; 2016). The challenges in scaling-up the microalgal culture include attaining optimal culture conditions, mixing, and achieving effective and efficient sampling and harvesting methods. The cultivation of *Pavlova lutheri* at 5–300 L for example, achieve the cell growth of 9.65 × 10^6^ cells/mL (0.35 g/L) in 300 L open-tank system, which is much less than the 12–14 × 10^6^ cells/mL (0.43–0.45 g/L) achieved in 250 mL reaction vessel (Shah et al. 2014a, b). The *Tetraselmis suecica*, *N. oculata, P. lutheri,* and *Isochrysis galbana* cultivation in 5 L photobioreactor (PBR) also achieve the highest biomass (0.62–0.96 g/L) and lipid content (31.6–42.2%), as compared to 0.45–0.72 g/L biomass and 24.4–38.5% lipid in 300 L open-tank (Shah and Abdullah 2018). The contents of palmitic, C16:0 (18.4%), oleic, C18:1 (11.3%), and pentadecanoic, C15:0 (8.16%) acids in *N. oculate*; and oleic, C18:1 (13.8.3%), palmitic, C16:0 (35.2%), and palmitoleic, C16:1 (23.3%) acis in *P. lutheri*, are the highest in 5 L PBR (Shah and Abdullah 2018). These may suggest the need to suit the mode of operation and algal cultivation for specific purpose.

**Table 1:** Production of biogas from the multi-algal co-digestion with EFB, POME and sludge inoculum (Ahmad et al. 2016)

Expt	Independent variables				Specific production of biogas rate (m^3^/kg COD/day)	Biomethane (mL CH_4_/ L POME/day)	CO_2_ (mL CO_2_/L POME/day)
Run	*N. oculata* (mL/mL POME)	*Chlorella* sp. (mL/mL POME)	*T. suecica* (mL/mL POME)	EFB (g/mL POME)			
Group A							
28	0	0	0	0.12	0.125	3539.0	3534.0
6	1	1	0	0.12	0.124	4651.9	2265.9
3	1	0	1	0.12	0.101	2765.2	2036.6
22	0	1	1	0.12	0.104	3541.6	1556.5
Group B							
17	1	0	0	0.06	0.095	3030.6	1730.0
10	0	1	0	0.06	0.121	3165.0	1883.4
8	0	0	1	0.06	0.099	2853.6	1550.6
23	1	1	1	0.06	0.108	3132.2	1853.6
9	1	1	1	0.06	0.111	3072.2	2272.0
Group C							
27	0	0	0	0	0.104	2540.0	2532.0
7	1	1	0	0	0.099	2579.8	2301.5
13	1	0	1	0	0.099	2778.9	1475.1
19	0	1	1	0	0.099	2353.0	1939.8
Group D							
18	2	1	1	0.12	0.097	4018.9	2079.5
2	1	2	1	0.12	0.099	2787.5	2272.4
26	1	1	2	0.12	0.108	1064.8	914.38
Group E							
24	2	1	0	0.06	0.124	1224.8	873.7
14	2	0	1	0.06	0.121	2123.6	1643.4
1	0	2	1	0.06	0.104	2224.8	1654.3
15	1	2	0	0.06	0.107	2787.5	1753.9
5	1	0	2	0.06	0.106	1229.4	1153.9
16	0	1	2	0.06	0.089	1026.7	934.6
Group F							
12	2	2	1	0.06	0.077	952.74	864.8
11	2	1	2	0.06	0.081	943.74	853.8
21	1	2	2	0.06	0.082	952.74	863.8
Group G							
4	2	1	1	0	0.108	3601.3	1543.3
20	1	2	1	0	0.096	2424.0	1984.9
25	1	1	2	0	0.088	870.5	803.4

**Table 2:** Fatty acids profile of *N. oculata and T. suecica* cultured in 10% POME in seawater (Shah et al. 2016)

Fatty acids (%)		*N. oculata*	*T. suecica*
Saturated fatty acid			
C12:0	Lauric acid	0.64	0.52
C14:0	Tetradecanoic acid	5.43	6.94
C15:0	Pentadecanoic acid	8.45	9.21
C16:0	Palmitic acid	28.22	36.48
C17:0	Heptadecanoic acid	2.31	3.62
C18:0	Stearic acid	7.44	8.33
C20:0	Eicosanoic acid	6.75	3.64
Total SFA		59.24	68.74
Monounsaturated fatty acid			
C16:1	Palmitoleic acid	9.37	5.81
C18:1	Oleic acid	5.77	6.45
Total MUFA		15.14	12.26
Polyunsaturated fatty acid			
C18:2	Linoleic acid	2.81	3.77
C18:3	Linolenic	4.56	5.11
C20:5	Eicosapentaenoic acid (EPA)	0.17	ND^a^
C22:6	Docosahexaenoic acid (DHA)	1.53	ND^a^
Total PUFA		9.07	8.88

^a^ND, not detected

### Cellulose extraction and modification

**Fig. 2:**
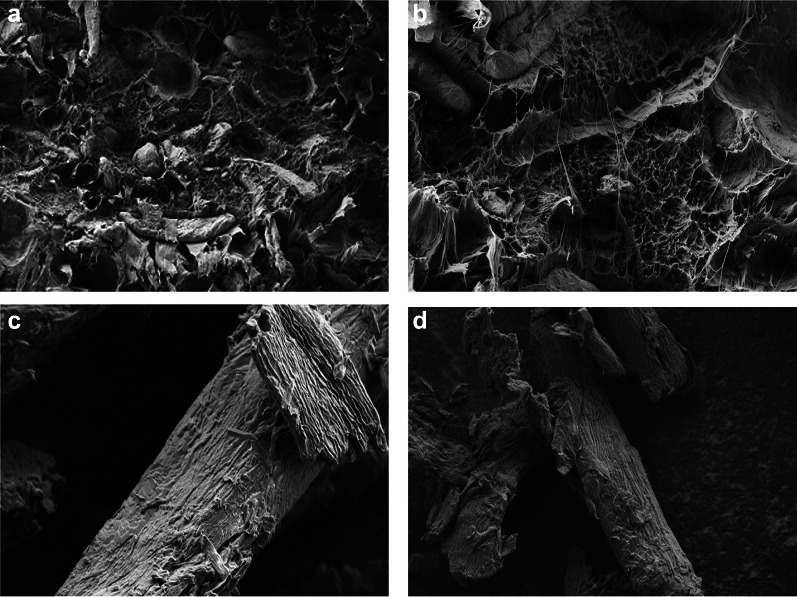
SEM of **a** 25% US-PEC/PP, **b** 25% AUTO-PEC/PP (reproduced from Abdullah et al. 2016b, with permission from Elsevier), **c** US-PEC-EDTA modified, **d** AUTO-PEC-EDTA modified (reproduced from Nazir et al. 2018a, b)

Globally, cellulose is the most abundant, cost-effective, and easily available natural polymer. It is the polysaccharides in the structure making up the plant cell walls (30–50% by weight). One of the largest and most well-known applications of cellulose is in the pulp and paper production (Mussatto and Loosdrecht 2016). An environmentally friendly technique has been developed for the isolation of purely extracted cellulose (PEC) from the EFB using ultrasonic (US) and H_2_O_2_ at 40 kHz and room temperature, to yield 49% PEC with 91.3% α-cellulose content and 68.7% crystallinity. The autoclave (AUTO) technique, with the combination of H_2_O_2_ and formic acid and more bleaching with H_2_O_2_ at 80 °C, yields 64% PEC with 93.7% α-cellulose and 70% crystallinity (Nazir et al. 2013). The polypropylene (PP) composites with 25% PEC loading fabricated by injection-moulding technique, attains the tensile intensity of 26.7–27.3 Mpa, without any addition of coupling factors (Fig. 2a, b). The surface engineering of PECs with Ethylene diamine tetra acetic acid (EDTA) treatment (Fig. 2c, d) has resulted in a lower degree of substitution (0.778–0.874), but with higher metal chelating ability. The high 232.9–236.7 mg/g Pb(II) sorption is attributable to the polydentate ligand (Nazir et al. 2018a, b). The Pb-loaded modified PECs also achieve high diesel desulphurization with 300–350 ppm sulphur removal, as compared to 80–110 ppm with the modified sorbents without Pb-loading (Nazir et al. 2018a, b).

### Bioproducts

**Fig. 3:**
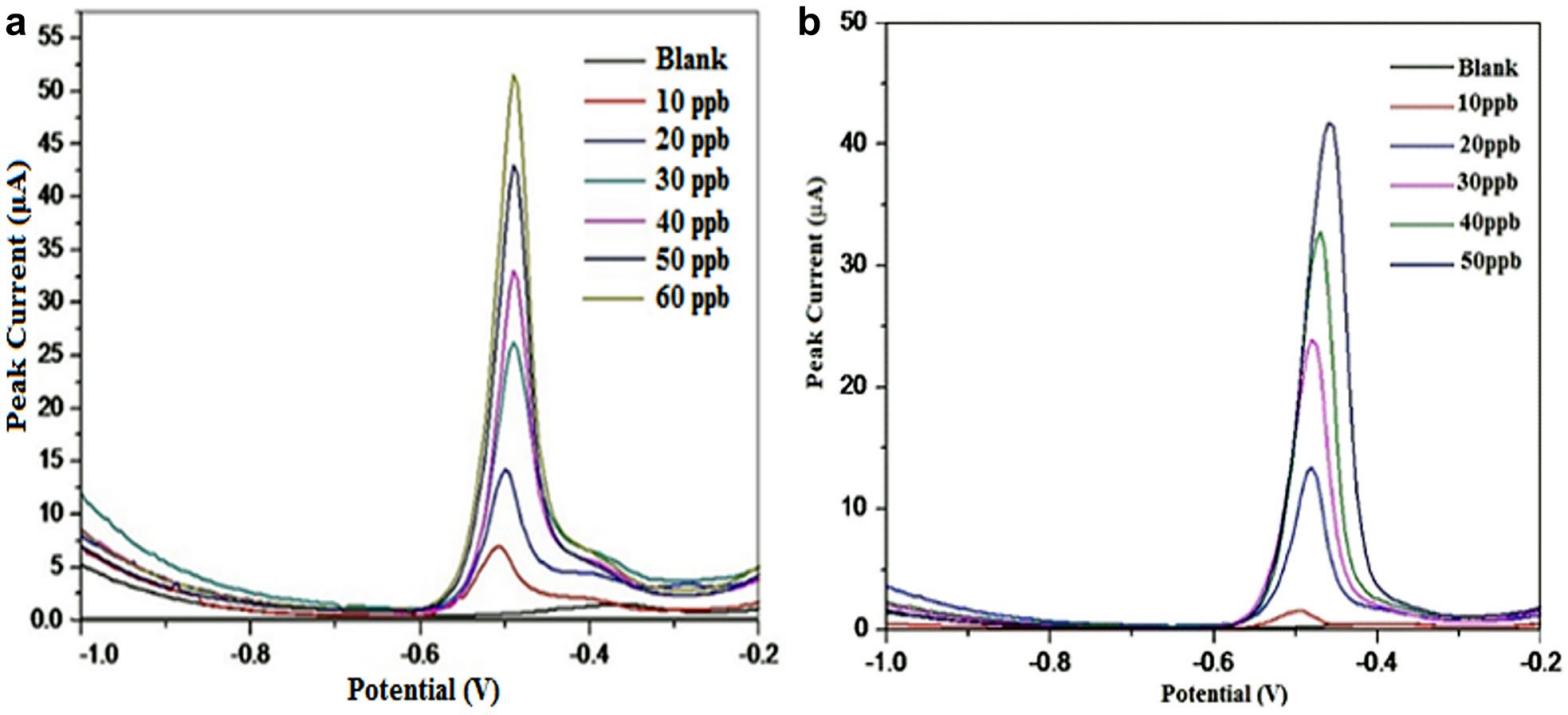
Cellulose-HAp-CME stripping voltammograms for Pb(II) ion detection in **a** digested blood serum (reproduced from Ajab et al. 2018, with permission from Elsevier), **b** digested POME (reproduced from Ajab et al. 2019, with permission from Elsevier)

Bioproducts extracted from biomass are non-toxic, biodegradable, and sustainable. The essential requirements for bio-proproducts from the biorefineries are the viability for large-scale production with higher degree of coupling between bioproducts and bioenergy co-generation. The cost of the raw materials, the cost of raw material processing, the market price of current and future bioproducts, marketability and practical use that suit the market needs (Budzianowski 2017), must be considered. The EFBs and the fibres have been used as soil conditioners or as absorbents to eliminate sulphur oxides (Sulaiman et al. 2011), turned into briquettes for solid fuels (Nasrin et al. 2008), or used in the manufactured furniture, packaging or building, electronics, and motorcar materials (Nazir et al. 2018a, b; Malaysian Palm Oil Council 2019). A novel, simple and low-cost preparation method has been developed for agro-based magnetic biosorbents based on the EFBs, *Ceiba pentandra*, and celluloses extracted from the EFBs to attain Pb(II) removal efficiencies of 97.7–99.4% from the aqueous system. The magnetic biosorbents can be reused 5 times for adsorption/desorption cycles with almost consistent high performance (Daneshfozoun et al. 2017). The fabrication of a novel oil palm-based cellulose-hydroxyapatite carbon composite electrode has successfully detected trace Pb(II) ions detection with the 0.095 ± 0.32 ppb limit of detection (LOD) and 0.32 ± 0.32 ppb limit of quantification (LOQ) in aqueous system (Ajab et al. 2020), which is comparable to 0.11 ± 0.36 ppb LOD and 0.36 ± 0.36 ppb LOQ, in blood serum (Fig. 3a) (Ajab et al. 2018), and 0.11 ± 0.37 ppb LOD and 0.37 ± 0.37 ppb LOQ, in POME (Fig. 3b) (Ajab et al. 2019).

### Biopharmaceuticals

#### Biocompounds

Biocompounds are the low volume but high-value products of a biorefinery. Table 3 shows different microalgal bioactive compounds which confer pharmacological and chemical novelty and bioactivities (El Gamal 2010). The secondary metabolites, though contribute only a fraction of the whole biomass, are involved in the basic machinery of life such as reproduction, growth, defence and the interaction with the surrounding environment, and therefore are vital for survival (Ianora et al. 2006; Cabrita et al. 2010). The major biocompounds such as lipids and fatty acids, carbohydrates and polysaccharides, proteins and enzymes, phenolics, carotenoids, vitamins and minerals, have exhibited different bioactivities.

**Table 3:** Microalgal bioactive compounds and the bioactivities

Compound	Source	Activity	References
1. Carotenoids β-Carotene	*Dunaliella salina*	Anticancer Anti-inflammatory Pro-vitamin A Antioxidant	(Ramos et al. 2011)
Astaxanthin	*Haematococcus pluvialis* *Chlorella zofigiensis*	Antioxidant Anti-inflammatory Anti-cancer	(Yuan et al. 2011; Liu et al. 2014)
Lutein	*Haematococcus pluvialis* *Chlorella protothecoides* *Chlorella pyrenoidosa*	Antioxidant Anti-inflammatory Anti-cancer	(De Morais et al. 2015; Cordero et al. 2011)
Violaxanthin	*Dunaliella tertiolecta* *Chlorella ellipsoidea*	Anti-inflammatory Anti-cancer	(Pasquet et al. 2011; Soontornchaiboon et al. 2012; Amaro et al. 2013
Zeaxanthin	*Chlorella Saccharophila* *Chlorella ellipsoidea*	Antioxidant Anti-inflammatory Antiproliferation	(Amaro et al. 2013; Singh et al. 2013)
Fucoxanthin	*Isochrysis sp.* *Odontella aurita* *Chaetoseros sp.*	Anticancer	(Amaro et al. 2013; Crupi et al. 2013)
2. Fatty acids			
Eicosapentaenoic acid (EPA)	*Tetraselmis sp.* *Chlorella minutissima*	Anti-inflammatory Anti-angiogenic	(Singh and Dhar 2011; Adarme-Vega et al. 2014)
Docosahexaenic acid (DHA)	*Tetraselmis* sp*.*	Anti-inflammatory	(Talero et al. 2015)
Docosapentaenoic acid (DPA)	*Nannochloropsis oculata*	Anti-inflammatory	(Talero et al. 2015)
Oleic acid	*Himanthalia elongate* *Nannochloropsis oculata* *Chlorella vulgaris*	Antimicrobial activity Antioxidant	(Plaza et al. 2010; Rajendran et al. 2014; Adhoni et al. 2016)
Linoleic acid	*Chlorella vulgaris* *Nostoc*	Antimicrobial activity Anti-cancer Antioxidant Anti-inflammatory	(Adhoni et al. 2016; Bhattacharjee 2016)
Palmitic acid	*Himanthalia elongate* *Spirulina platensis* *Tetraselmis* sp*.* *Dunaliella* sp. *Chlorella* sp*.*	Antimicrobial activity Anti-oxidant	(Plaza et al. 2010; Rajendran et al. 2014

### Lipids and fatty acids

Glycoglycerolipids such as digalactosyl diacylglycerol (DGDG), sulfoquinovosyl diacylglycerol (SQDG), and monogalactosyl diacylglycerol (MGDG) can be produced by microalgae. These lipids have significant bioactivities including in the prevention of tumour-promoting activities (Matsumoto et al. 2000), and anti-inflammation and immuno-suppressive activities (Bruno et al. 2005). Green algae have a varied range of C16 and C18 (unsaturated and saturated) fatty acids, and several species, although generally rare, have high amount of eicosapentaenoic acid (C20:5 n-3) fatty acid constituents. Chlorophyceae contains a mixture of fatty acids, similar to those found in the upper plants and oleaginous yeast. The active substance steaoryl-CoA in Chlorophyceae has resulted in a high level of C18 unsaturated fatty acids (Behrens and Kyle 1996). The polyunsaturated fatty acids (PUFAs) production from marine and freshwater algae has attracted attention (Wen and Chen 2003; Sijtsma and De Swaaf 2004) as the constituents such as eicosapentaenoic (EPA) and docosahexaenoic (DHA), naturally found in marine food chains, could be major therapeutic agents as anti-inflammation factors, and to tackle the incidence of cardiovascular disease and arthrosclerosis in humans (Moheimani and Borowitzka 2006). The omega-3 (ω-3) and omega-6 (ω-6) fatty acids are essential for tissue regeneration and these fatty acids cannot be manufactured. Both DHA and EPA could reduce the triglycerides and increase the high density lipoprotein (HDL) levels. Since the breast milk contains high amount of DHA and EPA, the newborns are also fed with the manufactured milk containing the ω-3 DHA as supplement (Guedes et al. 2011). Arachidonic acid (ARA), which belongs to the ω-6 PUFA, is important as a source of diet for the general good health of pregnant woman, newborns children and elderly people. In plants, ARA can be produced from linoleic acid, and linoleic acid in turn is synthesized from oleic acid (Tallima and El Ridi 2018). Thus, it is pertinent to promote oleic acid production as an essential microalgal fatty acid constituent. PUFAs also exhibit a defensive role against free radicals affecting the skin (Natrah et al. 2007). Another interesting group of lipids from macro and microalgae is sterol (Volkman 2005; Cardozo et al. 2007). Phyto-sterols extracted from microalgae have applications in production of therapeutic steroids, cosmetics, as functional foods for anti-cancer and anti-cholesterol activities (Volkman 2005; Francavilla et al. 2012).

### Carbohydrates and polysaccharides

Carbohydrate makes up 75% of the global biomass constituent. The sugar units could be used to store energy and other important functions in living organisms (Appelt et al. 2013). Polysaccharides are high value-added ingredients in cosmetics, foodstuffs, stabilizers, cloth, medicine, and emulsions (Arad and Levy-ontman 2010). The use of microalgal polysaccharides in the pharmaceutical industry is attributable to the ease of the compound isolation (Bhattacharjee 2016). Several natural or slightly pure polysaccharides extracted from different green, red, and brown algae have been studied for their anti-tumour properties (Ramberg et al. 2010), anti-bacterial, antioxidative, anti-inflammatory, and antiviral activities (Michalak and Chojnacka 2015). The major component in *I. galbana* and *N. oculata* polysaccharide extracts include glucose at 56.9 and 68.3%, respectively, while the second major compound is mannitol (38.8%) in *I. galbana*, and inositol (20.32%) in *N. oculata* (Hafsa et al. 2017). Nitrate concentration in the medium could affect starch accumulation in microalgae including *T. suecica* which is rich in intracellular polysaccharide (Kermanshahi-pour et al. 2014). Sulphate esters or sulphated polysaccharides from *C. vulgaris, Scenedesmus quadricauda*, and *Porphyridium* sp. (De Morais et al. 2015), could prevent viral infections such as encephalitis virus, herpes simplex virus 1 and 2 (HSV1, HSV2), haemorrhagic septicemia in salmonid virus, HIV, and swine fever virus (Smelcerovic et al. 2008; Amaro et al. 2011). The biological activities of sulphur polysaccharides is related to the formation of sugar, and the location and the degree of sulphurization (Kim et al. 2012). The polysaccharide GA3P (D-galactan sulphate, which is related to L-(+)-lactic acid), produced extracellularly by the toxic dinoflagellate *Gymnodinium* sp. A3, is a strong inhibitor of DNA topo I and topo II, whether or not there is the presence of lactate group (Umemura et al. 2003). It also inhibits colon cancer cell lines including HCC2998, KM-12, HCT-116, HCT-15, WiDr, and HT-29 (Talero et al. 2015).

### Proteins and amino acids

Different species exhibit different protein levels. The aqueous extracts of *N. oculata*, for instance, show higher protein level at 21.0 ± 0.2% as compared to *I. galbana* at 5.2 ± 0.17% (Hafsa et al. 2017). The normal C/N ratio of fresh water microalgae is 10.2, and a lower C/N ratio in some microalgal species, as compared to the terrestrial plants, could lead to a higher protein composition (Yen and Brune 2007). Nitrogen deficiency could increase the carbohydrate content by fourfold in *Tetraselmis subcordiformis* (Minhas et al. 2016). The intermediates of the carbohydrate metabolism such as pyruvate, a major precursor in aerobic glycolysis, in turn, could be used for the synthesis of amino acids such as leucine (Leu), alanine (Ala), and valine (Val). On the other hand, low carbohydrate content with significant amino acids such as Leu, Ala, Val, glutamate (Glu), and isoleucine (Ile), have been reported in *C. vulgaris* exposed to high CuCl_2_ concentrations (Zhang et al. 2014). Proteins from marine resources have unique characteristics such as the film and foaming ability, gel formation capacity and anti-microbial activity (Rasmussen and Morrissey 2007). *T. suecica* which contains large quantity and high quality intracellular protein content, is currently produced as aquaculture feed (Kermanshahi-pour et al. 2014). *Arthrospira* and *Chlorella* sp. which are rich in protein and amino acids are advantageous for use as dietary nutrients or as functional foods to inhibit tissue damage and diseases (Santhosh et al. 2016). The phycobiliproteins from red algae and cyanobacteria such as *Porphyridium* sp. and *Spirulina platensis*, have shown anti-inflammatory, hepatoprotective, immune, anti-oxidant and anti-cancer activities (Romay,Gonzalez,Ledon,Remirez,and Rimbau 2003; Zheng et al. 2011). The phycobiliprotein C-phycocyanin produced by *Spirulina platensis* induces the release of cytochrome *C* from mitochondria and triggers caspase-dependant apoptosis in HeLa cell lines (Li et al. 2006). The C-phycocyanin-mediated mitochondrial-dependant cell death has also been exhibited in the dimethylhydrazine (DMH)-induced colon cancer in the rat model (Saini et al. 2012).

### Phenolic compounds

Phenolic compounds such as flavonoids have nutraceutical properties, and lignans can be of great benefits in health and care products (Metsämuuronen and Sirén 2019). Polyphenols act as antioxidants during the transfer of single electron and during the transport of hydrogen atom (Goiris et al. 2012a). Many phenolics such as flavonoids, isoflavones, dihydrochalcones and flavonols are found in microalgae (Jahnke 1999; Natrah et al. 2007). Several phenolic compounds show anti-melanoma or anti-skin cancer activities (Talero et al. 2015), and antivirals, anti-carcinogenic, antimicrobials, anti-inflammatory or anti-tumour activities (El-Baky et al. 2009; Namvar et al. 2014). Phenols are important in the formation of anti-microbial compounds like cresol and ditol (Aiyegoro and Okoh 2010). High anti-microbial activities have been exhibited by the *N. gaditana*, *Tetraselmis* sp. and *Phaeodactylum tricornutum* ethanol extracts, attributable to the high amount of polyphenols at 32, 25.5 and 16.8 mg per gallic acid equivalent (GAE)/g biomass, respectively (Maadane et al. 2017). High phenolic content (> 3 mg GAE/g biomass) has been reported in the ethanol/water extracts of *Isochrysis, Phaeodactylum* and *Tetraselmis* sp. (Goiris et al. 2012b). *Chlorella*, which contains large amount of phenolics content, similar to the level in other plant sources, also exhibits high antioxidant activity at 58.73 mg GAE/g (Zakaria et al. 2017).

### Vitamins and minerals

Microalgae are important sources of essential vitamins such as tocopherol, ascorbic acid, B1, B2, B6, B12, nicotinic acid, and biotin; in addition to macro-minerals (K, Na, Mg, Ca) and micro-minerals (Zn, Fe, Mn, Cu) (Christaki et al. 2011). Vitamins such as β-carotene (pro-vitamin A), tocopherol, folic acid and thiamine are found in higher concentrations in some marine algae as compared to the other traditional foods considered as rich sources of these vitamins. *Dunaliella tertiolecta* and *Chlorella stigrnatophora* exhibit the highest concentrations of vitamin A, while *T. suecica* and *D. tertiolecta* for thiamin (Fabregas and Herrero 1990). Besides synthesizing β-carotene, *D. salina* also synthesizes pyridoxine, thiamine, nicotinic acid, tocopherol, biotin and riboflavin (Santhosh et al. 2016). *Spirulina and Chlorella* are rich with vitamins B complex, particularly B12, that is essential for the production and renewal of blood cells (De Morais et al. 2015). Diatom *Haslea ostrearia* (Navicula) is especially rich in vitamin E, while *Porphyridium cruentum* is rich with vitamins C, E (tocopherols) and β-carotene. *T. suecica* and *D. tertiolecta* cultured under nitrogen deficiency conditions have been found to have the vitamin E production increased (Sathasivam et al. 2019). The production of bio-based vitamins within the biorefineries setting can be linked to the bioenergies co-generation. The production of vitamin B2 by the fermentation of vegetable oil with the microbes, for example, has been coupled to the anaerobic digestion of the residual biomass for biomethane generation (Budzianowski 2017).

### Carotenoids

In photosynthetic organisms such as plants and algae, carotenoids act as pigments for light-harvesting and protecting the photosynthetic machinery from excessive light and oxidative stress by scavenging the reactive oxygen species (ROS), singlet oxygen, and free radicals (Deeming-Adams and Adams 2002; Kelly and Strasser 2011; Skibsted 2012). The carotenoids consist of xanthophylls (containing oxygen) and the carotenes (pure hydrocarbons with no oxygen) and contribute significantly in the overall anti-oxidant activities of algae (Takaichi 2011; Goiris et al. 2012b). The xanthophylls produced by higher plants or green microalgae include antheraxanthin, violaxanthin, neoxanthin, zeaxanthin, and lutein. However, microalgae contain more xanthophylls such as astaxanthin, canthaxanthin, and loroxanthin, while both algae and diatoms can synthesize diadinoxanthin, fucoxanthin, and diatoxanthin (Barredo 2012). Carotenoids could stimulate the immune system and reduce the incidence of chronic diseases such as cancer, heart disease, arthritis, and early aging (Mojaat et al. 2008). Although synthetic carotenoids can be cheaper, there is a need to consider natural carotenoids as the latter may have less concern with regards to their biological functions and food safety for human consumption (Li et al. 2011). When compared to the synthetic type, the purified β-carotene from *D. salina* could induce apoptosis in the prostate cell lines (Jayappriyan et al. 2013). The asbestos workers or smokers administered with the synthetic β-carotene have been reported to face higher risk of getting cancer or cardiovascular problems (Omenn et al. 1996). Several species such as *Chlorella* sp., *T. suecica*, *Isochrysis,* and *Phaeodactylum* sp. contain high carotenoids (> 3 mg/g biomass) in the ethanol/water extracts, with the highest at 7.8 mg/g biomass, while the lowest (1.65 ± 0.10 mg/g biomass) is observed in *N. oculata* (Goiris et al. 2012b). The major carotenoids in *C. fusca* and *C. vulgaris* include lutein, β-cryptoxanthin and β-carotene, and the ratios are different with higher lutein is detected in *C. fusca* (69.54 ± 11.29 µg/g DW) and *β-*carotene (18.42 ± 9.2 µg/g DW) *in C. vulgaris* (Othman et al. 2017). *Dunaliella salina* has been identified as the major source of β-carotene, with over 14% of the dry biomass content (Jayappriyan et al. 2013), and astaxanthin content in *Haematococcus pluvialis* is reported at 1–8% of the dry biomass (De Morais et al. 2015). Astaxanthin can be utilized as a bio-nutraceutical in salmon farming (Budzianowski 2017).

### Bioactivities

#### Anti-cancer

**Fig. 4:**
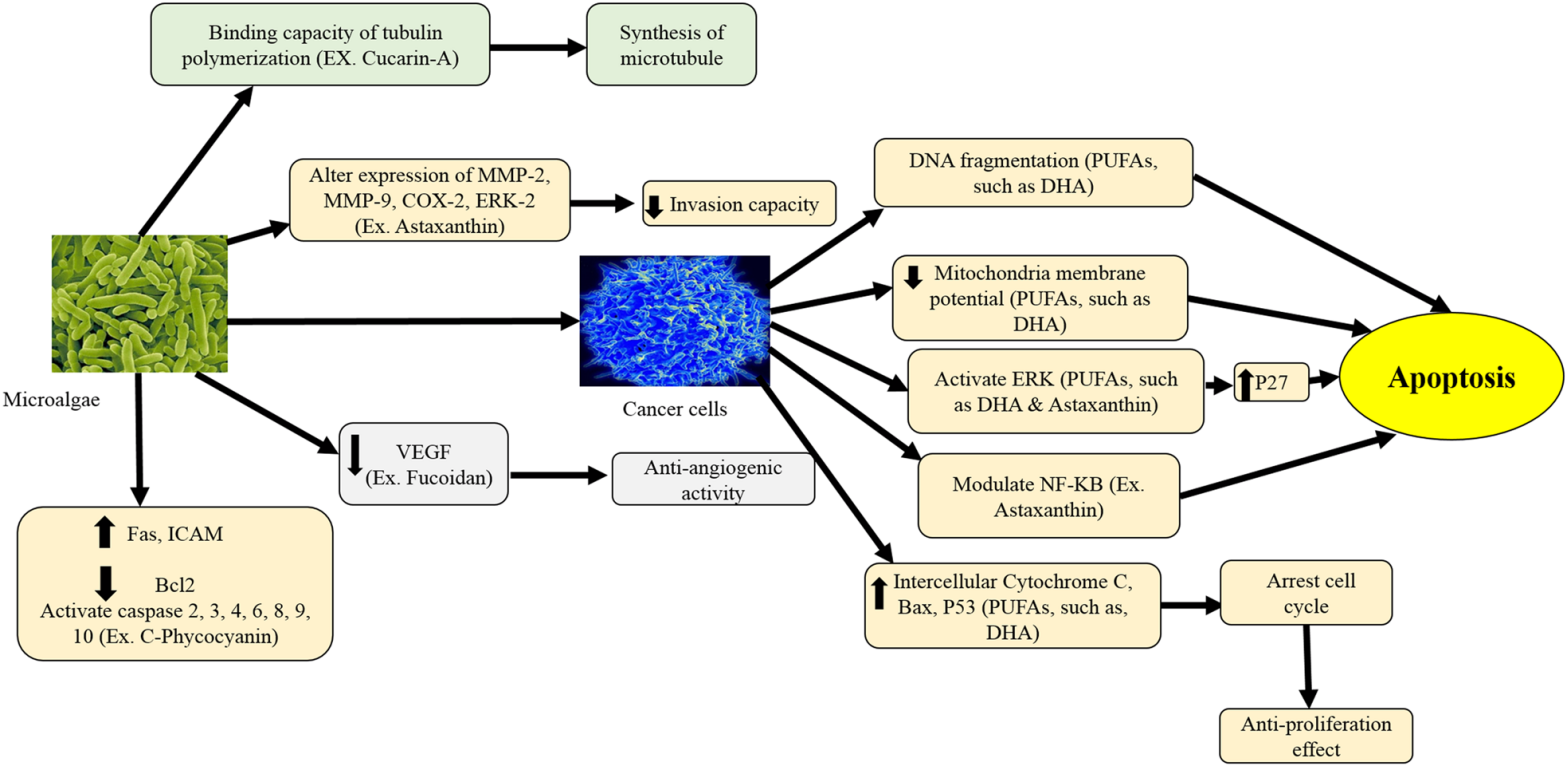
The possible anticancer mechanism of microalgae (modified from El-hack et al. 2019, Microalgae in modern cancer therapy: current knowledge. Biomedicine & Pharmacotherapy, 111:42–50)

Many anti-tumour agents derived from marine origin are from marine algae (Mayer and Gustafson 2008; Devi and Bhimba 2012; Monteiro et al. 2014; Sharif et al. 2014). The antioxidant and anti-inflammatory effects, and the dietary fibres may have contributed towards the anti-cancer activities of the marine algae (Kannan et al. 2010; Matharasi et al. 2018). Figure 4 shows the anti-cancer mechanism/s of microalgae (El-hack et al. 2019) including in the prevention of cancer cell growth, invasion, and metastases; reduced synthesis of microtubules; anti-angiogenic activity; and stimulation of programmed cell death in cancer cells (Farooqi et al. 2012; El-hack et al. 2019). The *Nannochloropsis oculata* extracts have shown no toxic effects on rats when administered orally, suggesting that the omega-3 oil present in the algal strain is not pathogenic (Kagana and Matulka 2015). The *Chaetoceros calcitrans* ethanol extracts (EEC) have shown higher anticancer activities against the breast cancer cells (MCF-7) than the normal MCF-10A cells, at the IC_50_ value of 3 ± 0.65 µg/mL after 24 h treatment (Nigjeh et al. 2013). The *C. sorokiniana* and *Scenedesmus* sp. have shown anti-cancer activity against murine tumour cell line L5178Y-R but no significant effects are reported on the viability of the normal murine thymus lymphocyte (Reyna-Martinez et al. 2018). The hot water-soluble polysaccharide compounds isolated from *Capsosiphon fulvescens* (chlorophycean algae) have shown the ability to stimulate apoptosis in AGS gastric cell lines (Kwon and Nam 2007). The *Sargassum muticum* (brown seaweeds) methanolic extract (SMME) exhibits the IC_50_ of 55 µg/mL and 22 µg/mL against MDA-MB-231 and MCF-7 cell lines, respectively (Namvar et al. 2013). The SMME can stimulate programmed cell death in human breast cancer cells whilst significantly reducing angiogenesis in chorioallantoic membrane (CAM) (Cabrita et al. 2010).

The *C. vulgaris* extracts have exhibited anti-cancer activity against hepatoma cell line (HepG2) at the IC_50_ of 1.6 mg/mL (Yusof et al. 2010; Lin et al. 2017), but the induction of apoptosis in *C. ellipsoidea* is 2.5 times higher than the *C. vulgaris* extracts (Cha et al. 2008; Lin et al. 2017). The *C. vulgaris* chloroform extracts have shown high cytotoxicity on the MCF-7 cells (IC_50_ of 89 μg/mL), but the activities are lower than those exhibited by the ethanol extracts of *Spirulina* and *Chlorella* (Syahril et al. 2011). The cytotoxicity of *Spirulina* extracts has been attributed to the presence of carotenoids, chlorophyll, and phycocyanin, and also polysaccharides (Hernandez et al. 2017). Novel compound sargaquinoic acid isolated from *S. heterophyllum* stimulates the apoptotic pathway in the metastatic MDA-MB-231 cells (IC_50_ of 67 μM) (De La Mare et al. 2012). Stigmasterol isolated from *Navicula incerta* also exhibits strong apoptotic inductive activity with great potential to be developed as anticancer therapeutics against liver cancer (Kim et al. 2014). The fucoidan compound extracted from *Fucus vesiculosus* exhibits cytotoxic effect against breast cancer in vitro and in vivo (Xue et al. 2012), and the crude fucoidan has significantly reduced the 4T1 cell numbers (a highly metastatic breast cancer cell line from mouse model) (Moussavou et al. 2014).

### Anti-oxidant

Various bioactivities such as anti-atherosclerotic, anti-inflammatory, anti-arterial and anti-carcinogenic activities can be linked to their anti-oxidant activities (Abdullah et al. 2017a). Synthetic antioxidants like butylated hydroxytoluene (BHT) or butylated hydroxyanisole (BHA) are carcinogenic (Namiki 1990; Pokorn 1991). Natural antioxidants are more appropriate for human consumption and the search for the new sources as viable alternatives to the synthetic ones has gained traction (Maadane et al. 2015). Natural antioxidants traditionally are from the plant origins, but the production from plants is expensive and time-consuming (Azim et al. 2018). Microalgae as a new source of antioxidant is attractive due to its relatively rapid growth and high content of secondary metabolites (Table 4) (Bule et al. 2018). As photosynthetic organisms, microalgae undergo continuous exposure to high oxygen concentrations and light, which stimulate the activation of free radicals and other oxidizing agents. Lack of damage to the microalgal structure suggests that their anti-oxidative mechanisms are effective for protection from the ROS and oxidative stress. Microalgae are rich in natural antioxidants such as tocopherols (Zubia et al. 2009), palmitoleic acid, linolenic acid, cyanovirin, phycocyanin, oleic acid, vitamin E and B12, lutein, zeaxanthin, phlorotannins, carotenoids, ascorbic acid and β-carotene (Harun et al. 2010). The anti-oxidant defence mechanisms may also involve the antioxidant enzymes (catalase or superoxide dismutase) and the low molecular weight antioxidants (LMWA) (such as glutathione, carotenoids, phenolics, ascorbate, and tocopherol) (Goiris et al. 2012a).

**Table 4:** Algal bioactive compounds (modified from Michalak and Chojnacka 2015)

Biologically active substances	Activity
Polyphenol Carotenoids Polysaccharide	Anti-tumour
Protein Mycosporine-like amino acid Glutathione Polyphenol Polysaccharide Polyunsaturated fatty acids (PUFAs) Carotenoids Tocopherol Ascorbate	Antioxidative
Protein Polyphenol Polysaccharide Chlorophyll and carotenoids PUFAs	Anti-bacterial
PUFAs Chlorophyll and carotenoids Terpenes Phenols	Anti-fungal
Protein Carotenoids Polysaccharide Sterols Polyphenol	Anti-inflammatory
Olyphenol Carotenoids Polysaccharide Diterpenes Protein	Anti-viral

The inhibition of cancer by algal extracts has been attributed to its antioxidant activities (Miranda et al. 1998). Based on β-carotene-linoleic acid bleaching method, different microalgal extracts have exhibited antioxidant activities such as *Oscillatoria* sp. hexane extract (97.7%), *S. platensis* ethyl acetate and water extract (93.6 and 90.1%, respectively), and *S. obliquus* chloroform extract (92.4 ± 0.3%), which are comparable to the standard synthetic antioxidant, BHT (97.7 ± 0.3%), but much higher than the ascorbic acid (AscA) (25.5 ± 0.2%) (Abdullah et al. 2017a). The DPPH (2,2-diphenyl-1-picrylhydrazyl) scavenging method exhibits low/moderate anti-oxidant activities ranging from 26.3 ± 0.7 to 69.1 ± 0.4%, as compared to the 85.8 ± 0.1% BHT and 94.6 ± 0.1% AscA (Ali et al. 2014a). The sensitivity of the assay methods is therefore pertinent to interpret the antioxidant activities and their correlations to the metabolite contents such as the phenolics compounds (Ali et al. 2014b). The methanolic extracts of *N. oculata* exhibit the highest DPPH radical scavenging activity at 400 µg/mL (21.68 ± 1.41% inhibition) as compared to the ethyl acetate extracts with 39.03 ± 0.97% inhibition (Ebrahimzadeh et al. 2018). *N. oculata* extracts exhibit antioxidant activities with the IC_50_ values between 4.93–7.31% (Custódio et al. 2015), while the water-soluble polysaccharides extracted from *N. oculata* and *I. galbana* at 10 mg/mL, show antioxidant activities of 59.07% and 41.45%, respectively (Hafsa et al. 2017). The *D. salina, Tetraselmis chuii* and *Isochrysis galbanaas* methanolic extracts at 50 ppm, as established by the DPPH assay, suggest that the *I. galbana* clone *Tahiti* exhibits the highest activity with 61.64% of free radicals inhibition, followed by *D. salina* (58.45%) and *T. chuii* (52.58%) (Widowati et al. 2017). *Chlamydomonas reinhardtii* and *C. vulgaris*, respectively, show high antioxidant activity at 1000 μg/mL with 83.38 and 92.57% DPPH free-radical scavenging activity, and 82.74 and 97.9% of the total anti-oxidant activity (Jayshree et al. 2016).

### Anti-microbial

Due to the increased phenomenon of antibiotic-resistant bacteria globally, there is an urgent need to develop a new and potent agent against infectious diseases (Sanmukh et al. 2014). The allelopathic compounds which can be produced by some algal species such as *Tetraselmis* sp., have the ability to decrease the growth of the competitor and predator microorganisms (Makridis et al. 2000). The anti-microbial activity of *Tetraselmis* sp. extracted by methanol, diethyl ether and hexane have been studied by using agar disc diffusion method against Gram-negative bacteria (*S. aureus, Bacillus subtilis, Pseudomonas aeruginosa*), Gram-positive bacteria (*Proteus vulgaris, E. coli*) and fungal pathogens (*Aspergillus niger, Aspergillus fumigatus,* and *C. albicans*). The higher inhibition zone is achieved with the methanolic extracts against *E. coli* (16 mm) and *S. aureus* (15 mm), and against the fungal pathogens *A. niger* (7 mm). All *Tetraselmis* sp. extracts however exhibit no effects against *P. vulgaris*, *A. fumigatus* and *C. albicans*. These anti-bacterial activities are attributed to the bioactive compounds in the methanolic extracts with promising pharmaceutical applications (Matharasi et al. 2018). The methanol and chloroform extracts of 3 seagrasses, with the methanolic extracts being the most active ones, inhibit the growth of all pathogenic bacteria tested such as *S. aureus*, *Vibrio cholerae*, *Shigella dysentriae*, *S. bodii*, *S. paratyphi*, *P. aeruginosa* and *Klebsiella pneumoniae* (Kannan et al. 2010). On the other hand, the hexane and methanolic crude extracts of *N. oculata*, *T. suecica* and *Chlorella* sp., in co-application with silver nanoparticles (AgNPs), at the 1.5:1 ratios (AgNPs:Microalgal extracts (w/w)), exhibit more consistent and stronger activities against *Bacillus subtilis*, *Streptococcus uberis*, and *Salmonella* sp., than the microalgal crude extracts alone. The AgNPs–*T. suecica*–HEX and MET, and the AgNPs–*Chlorella* sp.–HEX, at the 1.5:1 ratios, also exhibit strong activities against *K. pneumonia* (Hussein et al. 2020b). The antimicrobial activities, as exhibited by the extracts from the solvents of low to moderate polarity, may be related to the fatty acids contents, phenolic compounds and carotenoids. However, the extracts from the more polar ethanol solvent, of *N. gaditana*, *D. Salina*, *Dunaliella* sp., *Isochrysis* sp. and *P. tricornutum*, have also shown inhibitory activities against *P. aeruginosa* and *Escherichia coli* with a minimum inhibitory concentration (MIC) at 2.6 to 4.3 mg/mL. The *N. gaditana* extracts show antimicrobial effects on *Staphylococcus aureus*, with the highest anti-fungal activity against *Candida albicans* at the MIC of 4.0 mg/mL (Maadane et al. 2017). Infact, the methanol, chloroform, diethyl ether and ethanol extracts of *C. vulgaris* have all shown anti-bacterial activity against the Gram-negative (G^−ve^) and Gram-positive (G^+ve^) human pathogenic bacteria (Dineshkumar et al. 2017). However, the degree of inhibition and activities may be different. For example, the effective inhibition zone of *Chlamydomonas reinhardtii* extracts against G^−ve^ bacteria (*P. aeruginosa*, *S. aureus,* and *E. coli*) are higher than the *C. vulgaris* extracts (Jayshree et al. 2016). Hence, the methods of extraction and the solvent systems used are of great importance to ensure that the compounds with antimicrobial/allelopathic activities could be isolated and identified.

### Chemical profiling and analyses

To efficiently utilize the bioproducts for its intended applications, it is pertinent to understand the diversity of organic compounds as precursors, “lubricants” or end-products in living organisms (Rojas et al. 2014). As the final products of the cellular regulatory mechanisms, the types and levels of compounds of a biosystem can be considered as the final response to any environmental or genetic perturbations (Fiehn 2002). The primary metabolites, including proteins, carbohydrates, nucleic acids, and fats, are essential and have wide classification and distributions (Hanssen 2014). Secondary metabolites, in contrast, are not dynamically produced by general metabolic pathways, have limited classification and distribution, and may be restricted to a particular species or genus (Gouvea et al. 2012; Sacristán-soriano et al. 2012). The challenge to get a snapshot of the biological responses in time and space is in having to analyse a huge array of metabolites and structures, and to develop optimal analytical procedures and measurements (Gomez-casati et al. 2013). The comprehensive and high quality analysis of metabolite mixtures is called metabolomics which has emerged as a useful methodology in functional genomics to understand the complex molecular interactions in biological systems (Hall et al. 2002; Gomez-casati et al. 2013). The developments have been much assisted by the advancement in the computing techniques and speed which allow the interpretation of metabolic data in relations to the metabolic pathways (Wishart 2007, 2011). With improved sensitivity and specificity of small molecule detection, identification and measurement of complex metabolic profiles in the biological specimen have led to simultaneous measurement of tens and hundreds of metabolites in a single sample (Psychogios et al. 2011; Bouatra et al. 2013). It has important applications in food technology, pharmacology, microbial biotechnology, drug and enzyme discovery, toxicology, plant biotechnology, and systems biology (Gomez-casati et al. 2013) (Additional file 1: Fig. S1). Metabolomics is a valuable tool to analyse herbal product composition, identify active metabolites and classify the specimens on the basis of different metabolites due to the environmental and genetic variations (Urso et al. 2018). In oncology, metabolomics has potential for evaluation of early cancer detection and for cancer diagnosis, prognosis and therapeutic efficacy; to predict and identify pharmacodynamics marker of any drug effects; and to provide a link between metabolic profiling and molecular imaging techniques to enable non-invasive discrimination of metabolic markers in vivo (Spratlin et al. 2009).

The development of advanced technology to separate and detect more metabolites allow aqueous and lipid-based metabolites to be evaluated simultaneously (Sas et al. 2015). The TLC/HPTLC (high-performance thin-layer chromatography) finger printing provide useful information on the marker chemical compounds and could help in the quality control and monitoring of some species (Murugesan and Bhuvaneswari 2016). The mass spectrometry (MS), often used alongside either the liquid chromatography (LC) or gas chromatography (GC), is one of the most common analytically sensitive techniques in metabolomics (Urso et al. 2018). The GC or LC separates the components of the mixture while the MS analyses each component separately and defines the structure of the compounds depending on the mass-to-charge ratio in the charged molecules (Arora and Kumar 2017). The GC–MS focuses primarily on the volatile metabolites and the derivitizable compounds with low polarity and which are more stable (Stringer et al. 2016), such as sugars, amino acids, sugar alcohols, organic acids, and polyamines. This may lead to a fairly inclusive coverage of the primary metabolites in the central pathways (Obata et al. 2013). The LC–MS could detect nanomolar concentrations, without any need for sample derivatization. The most common soft ionizer is the electrospray ionization (ESI) which is usually associated with the LC–MS, and it is the best to analyse the compounds with ionic functional groups (Matich 2018). Both LC–MS and GC–MS methods complement each other (Matich 2018). Nuclear magnetic resonance (NMR) spectroscopy is ideal for primary macro-analysis at the metabolism level of the organism (Pan and Raftery 2007). Though less sensitive than the MS method, NMR is non-invasive, non-selective and with easy sample preparation steps. The metabolomics based ^1^H NMR has been used extensively for non-targeted analysis to determine the relationship between the metabolites detected in the spectra of the biological extracts, to their bioactivities (Azizan et al. 2018). Each metabolite has a special NMR spectrum that exhibits the environment of each proton. These vibrations are further broken down by the interaction with the protons of the neighbouring carbon atoms. The area below the peak is directly proportional to the metabolite concentration, which can be measured using an appropriate internal standard such as 2,2-dimethyl-2-silapentane-5-sulfonate (DSS) (Stringer et al. 2016). The NMR coupled with the multivariate data analysis (MVDA) methods could be used to develop and obtain maximum targeted metabolites in a short time. Advances and interrelated analyses based on NMR and MS could ultimately complement each other and shift the study on the metabolism to a new paradigm (Azizan et al. 2018).

The metabolomics studies of the biological samples typically consist of five different steps (Additional file 1: Fig. S2): (1) experimental design; (2) sample preparation; (3) separation and detection of metabolites; (4) data processing; and (5) bioinformatics analysis (Sas et al. 2015). The essential component of NMR-based and MS-based studies is the suppression/extraction of the metabolites, collection of data and processing and analysis of data or the chemometric tools (Urso et al. 2018). Both NMR and MS analyses show a higher amount of chlorophyll and other polar compounds in the chloroform/methanol extracts as compared to the hexane extracts. In a smaller (500 mg) solid phase extraction columns for over 50 mg extract weights, *N. oculata* has shown higher chlorophyll contents while *T. suecica* shows significant PUFA as compared to the other microalgae (Danielewicz et al. 2011). The ^1^H-NMR spectroscopy of *Wolfipori cocos* detects 33 different chemical metabolites in D_2_O, containing 11 organic acids, 13 amino acids, 3 sugar alcohols, 2 sugars, and 1 nucleoside. *W. cocos* could therefore be cultivated to extract large quantities of bioactive compounds for use in nutraceutical and pharmacological applications (Oh et al. 2018). The various metabolites in the *Fragaria vesca* extracts, most of which have already been reported in the *Fragaria ananassa* species, could be classified as belonging to the flavonoids, catechin, oligomirates and igitaine (Kårlund et al. 2015). The LC–MS data has been correlated to the antioxidant activity for different extract as determined by the trolox equivalent antioxidant assay (TEAC) (Urso et al. 2018). The *A. kurrat* methanolic extract shows higher anti-oxidant activity as well as cytotoxic activity against human colon carcinoma (Caco-2) and HepG2 based on the neutral-red test. The main components of the methanolic extract and ethyl acetate fraction, as determined by the HPLC–ESI-MS analysis, have confirmed the presence of phenolic acids and flavonoid glycosides which may contribute towards the anti-cancer activity (Abdel-Hady et al. 2018).

### Drug delivery and nanomedicine

**Fig. 5:**
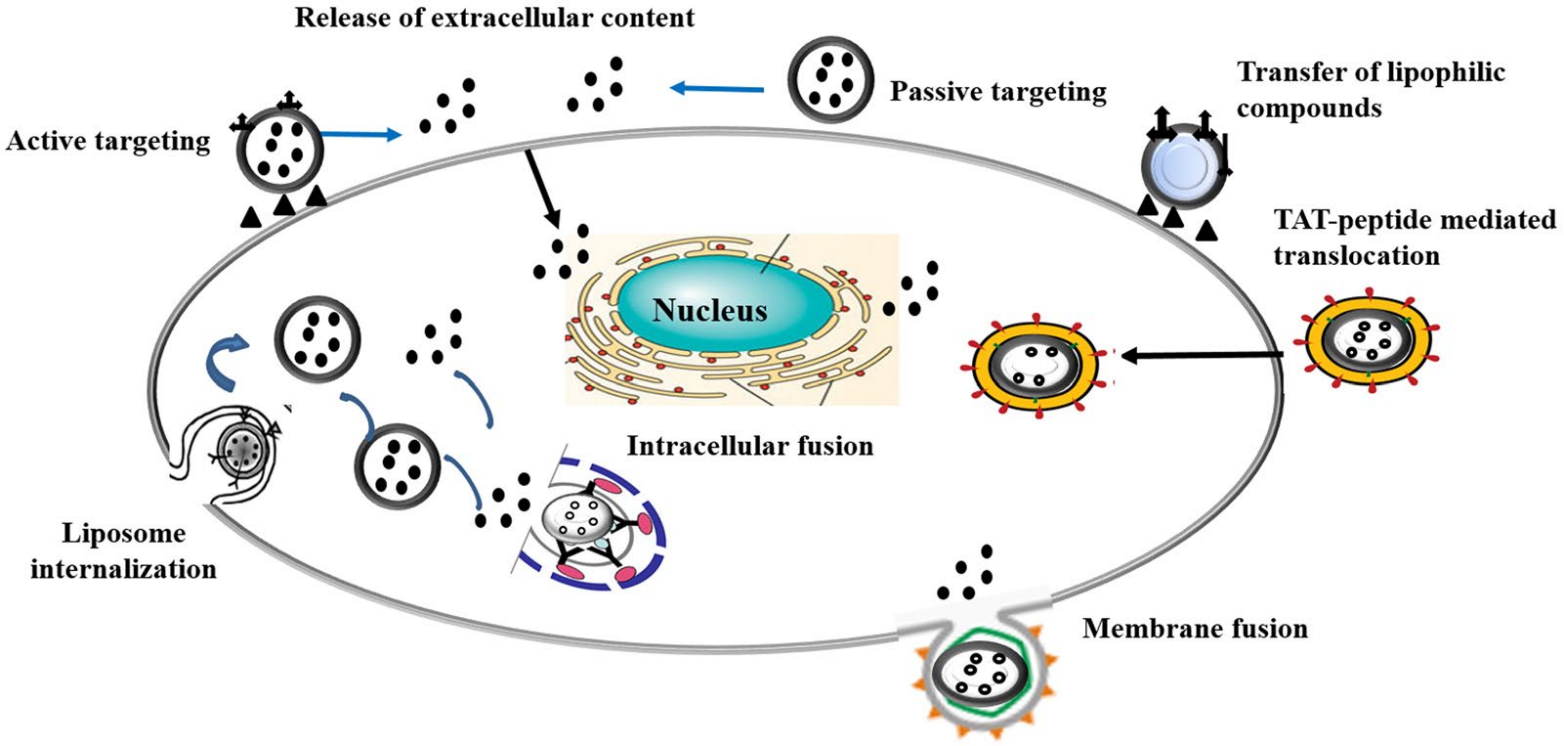
Liposome as a drug delivery system (TAT—transactivator of transcription) (modified from Shaheen et al. 2006)

Integrating the algal biocompounds production with nanomedicine could lead to a more affordable healthcare, and revolutionize cancer therapeutics with minimal or no side-effects (Abdullah et al. 2014; Gul-e-Saba and Abdullah 2015; Hussein et al. 2020a). The microalgal crude extracts (MCEs) and bioactive compounds with metal nanoparticles such as the AgNPs, have potentials in cancer treatment and for theranostics applications. The AgNPs are medically related metallic NPs with different degrees of cytotoxicity which can target specific diseases and cancer cells and tissue (Wicki et al. 2015). High cytotoxicity on MCF-7 and 4T1 breast cancer cell lines but without any cytotoxicity on the non-cancerous Vero cells, have been shown by the co-application of AgNPs–MCEs–chloroform (CHL) (Table 5) (Hussein et al. 2020a, c). The microalgal metabolites even reduce the cytotoxic effects of elevated AgNPs against the non-cancerous Vero cells whilst retaining the cytoxocity against the cancerous MCF-7 and 4T1 cells (Hussein et al. 2020d). This could open up a new avenue for the use of natural products from microalgae with nanoparticles for high-value pharmaceutical applications. As the active pharmaceutical ingredients (API) in chemotherapy can be very toxic to both normal and cancer cells, the microalgal extract-drug formulation (Hussein et al. 2020a), or liposomes as drug delivery systems (Fig. 5) (Shaheen et al. 2006), could specifically target the cancerous cells and spare the healthy cells or tissues. These may be necessary for treatment efficacy at the rate based on the needs of the body and targeted site. Liposomes consist of phosphatidylcholine-enriched phospholipids, and the mixture of the phosphorus chains with surfactant properties. The vesicle system and the bilayer vascular structures form spontaneously when phosphorus is dispersed in water, with colloidal domains of the non-active cholesterol, glycolipids, long-chain fatty acids, sphingolipids, and proteins, and the drug molecules (Tiwari 2013; Suriyakala et al. 2014). A few drugs have been formulated as lipids to improve their treatment, and liposomes can be used as carriers of anticancer and antitumor drugs, antifungal drugs, analgesic drugs, gene therapeutics and vaccines (Yadav et al. 2017; Olusanya et al. 2018). The microalgal phospholipids (PL) for the liposomes production, provide a mixture of the hydrophobic compounds and ω-3 fatty acids, and can increase the DHA adsorption in infants, and reduce the cholesterol and the cirrhosis in mammalian models (Lawlor et al. 2017). The PLs extracted from *Galaxoura cylindriea*, *Laurencia popillose*, *Ulva fasciata*, *Taonia atomaria*, and *Dilophys fasciola* have exhibited higher anti-cancer activity against human liver and breast cancer cells in vitro at the IC_50_ values of 0.47 to 3.15 µg/mL, respectively. The PL of *U. fasciata* and *L. popillose* also show antiviral activity against simplex virus type 1 and antimicrobial activity against *B. subtili* and *E. coli* at the MIC of 40 µg/mL (Abd El Baky et al. 2014).

**Table 5:** IC_50_ values of AgNPs–MCEs–CHL at different ratios and duration of treatments on MCF-7, 4T1 and Vero, cell lines (modified from Hussein et al. 2020a, c)

Cell lines/co-application	24 h				48 h				72 h			
	1:1	1.5:1	2:1	1.5:3	1:1	1.5:1	2:1	1.5:3	1:1	1.5:1	2:1	1.5:3
1. MCF-7												
AgNPs–*N. oculata*–CHL	30.19 ± 0.24	19.95 ± 0.20	28.18 ± .15	60.25 ± 0.10	25.7 ± 0.20	15.13 ± 0.20	20.41 ± 0.20	44.66 ± 0.23	20.89 ± 0.20	10.47 ± 0.30	17.78 ± 0.26	33.88 ± 0.20
AgNPs–*T. suecica–*CHL	31.62 ± 0.10	15.84 ± 0.20	14.12 ± 0.25	45.7 ± 0.19	30.19 ± 0.30	13.8 ± 0.10	13.8 ± 0.20	41.68 ± 0.10	25.11 ± 0.29	7.63 ± 0.29	6.60 ± 0.30	38.01 ± 0.23
AgNPs–*Chlorella* sp.–CHL	47.86 ± 0.10	41.68 ± 0.21	24.54 ± 0.25	52.48 ± 0.18	29.5 ± 0.10	37.15 ± 0.24	20.41 ± 0.24	50.11 ± 0.21	19.05 ± 0.20	33.88 ± 0.16	14.45 ± 0.20	47.86 ± 0.16
2. 4T1												
AgNPs–*N. oculata–*CHL	85.11 ± 0.03	85.11 ± 0.10	63.09 ± 0.10	–	75.85 ± 0.10	81.28 ± 0.04	54.95 ± 0.04	–	66.06 ± .08	79.43 ± 0.07	52.7 ± 0.15	–
AgNPs–*T. suecica–*CHL	95.49 ± 0.06	66.06 ± 0.10	66.06 ± 0.09	–	93.32 ± 0.04	66.06 ± 0.05	60.25 ± 0.10	–	79.43 ± 0.08	56.23 ± 0.10	53.7 ± 0.06	–
AgNPs–*Chlorella* sp.–CHL	89.12 ± 0.09	58.88 ± .08	63.09 ± 0.08	–	87.09 ± 0.03	56.23 ± 0.10	60.25 ± 0.10	100 ± 0.08	79.43 ± 0.06	48.97 ± 0.06	50.11 ± 0.06	100 ± 0.03
3. Vero												
AgNPs–*N. oculata–*CHL	–	–	–	–	–	–	–	–	–	–	–	–
AgNPs–*T. suecica–*CHL	–	–	–	–	–	–	–	–	–	–	–	–
AgNPs–*Chlorella* sp.–CHL	–	–	–	–	–	–	–	–	–	–	–	–

Data expressed as mean ± standard deviation (*n* = 3). Statistically significant difference between microalgae extracts, ratio and time exposure. The symbol “– “ indicates no IC_50_ estimate

### Global sustainable development goals (SDGs) and life cycle analysis (LCA)

**Fig. 6:**
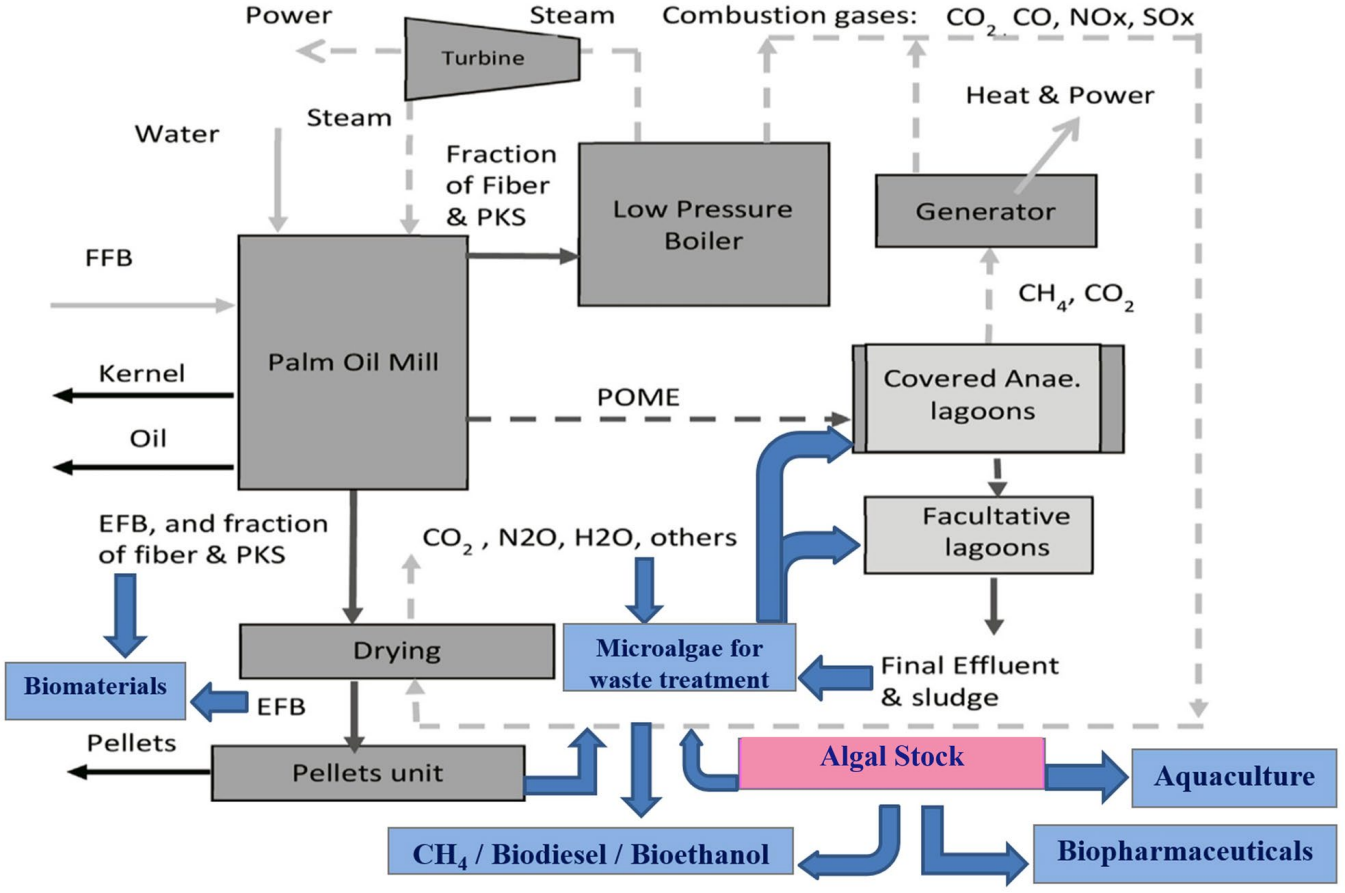
The integrated algal and oil palm biorefinery (modified from Garcia-nunez et al. 2016; Abdullah and Hussein 2020)

The concept of an integrated biorefinery based on palm oil mill (Garcia-Nunez et al. 2016) and co-cultivation of microalgae (Fig. 6) (Abdullah and Hussein 2020) should meet the SDG 7 on affordable and clean energy, SDG 8 on decent work and economic growth, and SDG 9 on the industry, innovation, and infrastructure. The indirect impacts include in reducing inequality (SDG 10), developing sustainable cities and communities (SDG 11), and in practising responsible production and consumption (SDG 12) (About the Sustainable Development Goals 2019). The key performance indicators should be based on the combination of economic performance indicator, with the environmental and social impact indicators. The LCA-based environmental impact assessment is performed with eco-efficiency analysis to evaluate the trade-offs between economic output, environmental impacts (such as pesticides, fertilizer use, field emissions, field operations or irrigation) and resource utilization (energy, water, land or materials) (Begum and Saad 2013; Ng and Ng 2013; Ng et al. 2013, 2015), and the associated problems in relation to the quality of air, water and soil; management of wastes and waste water; GHG emission; maintenance and preservation of biodiversity and wildlife; and energy efficiency (Pérez et al. 2017). The scope and goal of an LCA are the two essential stages in which the system boundaries and the system definition depend on. The goal will determine the design, operation, and policy to be formulated and executed. For effective design and operation, the system definition must address the global perspective. A simple flowchart on biofuels production may exhibit the policy formulated, but for the policy to be an essential part of the LCA framework, system boundaries must be well-defined according to the scope and the goal set. For instance, if bioethanol production is analysed within the boundary of the well-to-tank (WtT) system, the engine's fuel combustion may have no direct impact in the LCA. However, the outcomes will be different, if the comparison of the bioethanol production is made with different fossil fuels or their mixture, within the same boundary system (Rathore et al. 2016). The economic and physical distribution based on energy and carbon content, mass (wet or dry), economic value, and system growth, are used to differentiate between the environmental load of production or process, and also to reflect the many functions within the same process (Rathore et al. 2016).

Palm oil accounts for one-third of the total vegetable oil production worldwide (Zahan and Kano 2018). The use of palm oil biodiesel is advantageous as its combustion does not add carbon dioxide, but just returning the carbon dioxide obtained earlier from photosynthesis, back into the atmosphere. The oil palm tree continues to absorb carbon dioxide throughout its 25–30 years of productive lifespan. The areas planted with oil palm in Malaysia also constitute only 2% of the land area globally dedicated to the oilseed crop planting (Basiron 2007). The economic viability of palm oil as a primary feedstock is attributed to the high oil content, low market price, abundant of resources and availability, and high productivity from lower farming areas as compared to the other oilseed crops (Zahan and Kano 2018). Based on the LCA, biodiesel from palm can decrease the GHG by 46–73% as compared to the diesel. The major environmental impacts are caused by the nitrogen-fertilizer production and its use in the oil palm plantation, and the emissions from the POME-treated ponds (Silalertruksa and Gheewala 2012a). The LCA carried out on the GHG emissions from the oil palm industry suggests the contributions from the Land-Use Change (LUC) for new areas of plantation, cultivation and harvesting, transportation, milling processes, conversion and processing into biodiesel and by-products, waste management and biodiesel usage (Silalertruksa and Gheewala 2012b). There are opportunities for more efficient utilization of fibres and shells, and biomethane from the wastewater treatment to improve the net energy ratio (NER) and net energy balance (NEB) (Pleanjai and Gheewala 2011). In the case of microalgal cultivation using raceway ponds and PBRs with only the production of biodiesel; and with both the production of ω-3 fatty acids and biodiesel, the LCA suggests for the capital and operating cost reduction of at least 50% to make it profitable, even with higher revenue from ω-3 fatty acid production (Sawaengsak et al. 2014).

The challenges to achieve commercial scale of microalgal biofuel are to identify high-lipid accumulating strain, to genetically engineer the strain with high tolerance to extreme environment, to achieve optimal engineering strategies for high productivity, efficient harvesting and product recovery, and support from the government (Rajesh Banu et al. 2020). The operation to enhance algal biomass productivity, and to achieve sustainable energy, water, nutrient/fertilizer, GHG emissions and land use across the entire value chain, must be systematically analysed based on techno-economic assesments and LCA (Laurens et al. 2017). There is a need to optimize the design for effective conversion into biodiesel with high productivity and high quality at minimal production cost and environmental impacts. A comprehensive strategy must be established to transport the wastes from the production sites and facilities; and to improve the reaction and conversion into biofuels, and the performance and effects on the engine. The improvements from LCA and sustainability studies should generate cheaper, environmentally friendly and high quality biofuels (Zahan and Kano 2018). Apart from the microalgae-oil palm biorefinery, the other routes may include the oil recovery, generation of biogas and sugar feedstocks and thermo-chemical processes (Shah and Abdullah 2017). The co-production of biofertilizers, biocomposites, commodity chemicals and aquaculture and animal feeds (Tran et al. 2017), biopharmaceuticals, EPA-rich oil and proteins (Chua and Schenk 2017; Shah and Abdullah 2017), are among the considerations to be made to further make the integrated algae–oil palm biorefinery, a more socially, environmentally and economically viable option. It is also high-time that the new concept, **HEESBA** (for **H**ealth consciousness; **E**nvironmental and safety awareness; **E**nergy sufficiency; **S**ocial inclusiveness; **B**usiness acumen; **A**daptability and agility), be embedded in the education curriculum, and practised in the government and private sector administration. “Hisbah”, which in Arabic means “Accountability”, should make Profit, Society and Wisdom as the key parameters in evaluating the Outcomes and Performances of any initiatives and endeavours. This is important to produce a new generation of politicians, policy makers, economists, business people, social scientists, engineers, industrialists, technocrats and the communities, that are more adaptable, and responsible, and with wisdom, could formulate the strategies and work out the solutions to the challenges confronting the society and the world, to meet the goals of economic and environmental sustainability, and social equitability.

## Conclusions

Integrated algal and oil palm biorefinery can pave the way towards achieving the global sustainable development goals to address the issues and impacts of climate change and green-house gas emission. The provision of clean and affordable energy, and the promotion of decent work and economic growth through green industry, innovation, and infrastructure, could at the same time reduce inequality, create sustainable cities and communities, which practise responsible production and consumption. These can be accomplished by reutilizing the oil palm residues, bioenergy co-generation, and waste treatment, with microalgal co-cultivation and conversion into value-added products. The extraction of high-value biochemicals and the development of biomaterial-based products could further improve the economic aspects. The algal cultivation as an alternative molecular pharming system could facilitate in the drug development based on algal natural compounds, either directly as drugs or as key molecules for the synthesis of biochemical drugs. The algal bioactive compounds could add value and improve the economic competitiveness of a biorefinery for a wide range of application including food industries, novel enzyme and drug discovery, microbial and plant biotechnology, pharmacology, toxicology, and systems biology. The life-cycle analysis incorporating economic performance, environmental and social impact indicators provide optimal options for a more informed and knowledge-based decision-making process.

### Supplementary Information

**Additional file 1: Fig. S1.** Applications of metabolomics (modified from Gomez-Casati et al. 2013, metabolomics in plants and humans: applications in the prevention and diagnosis of diseases. *BioMed Research International*, 1–11). **Fig. S2.** Flow diagram of the metabolomics studies (modified from Sas et al. 2015, Metabolomics and diabetes: Analytical and computational approaches. *American Diabetes Association*, 64:718–732).

## Data Availability

The data and materials will be made available upon request.
