# Supplementary figures and images for: Integrated algal and oil palm biorefinery as a model system for bioenergy co-generation with bioproducts and biopharmaceuticals
Source: Bioresour Bioprocess. 2021 May 20;8(1):40. doi: 10.1186/s40643-021-00396-0 (PMC10992906; doi:10.1186/s40643-021-00396-0)

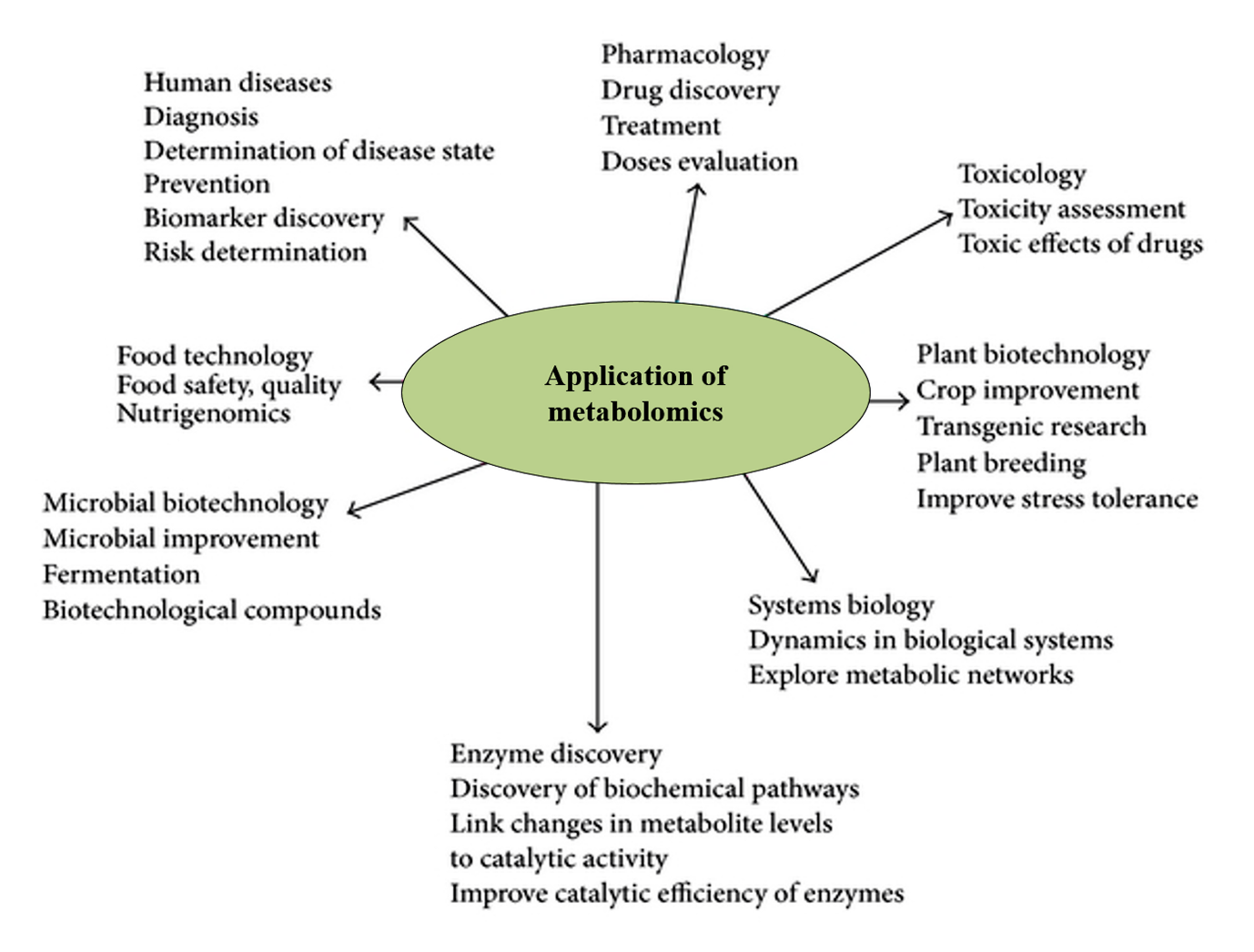


Figure S1


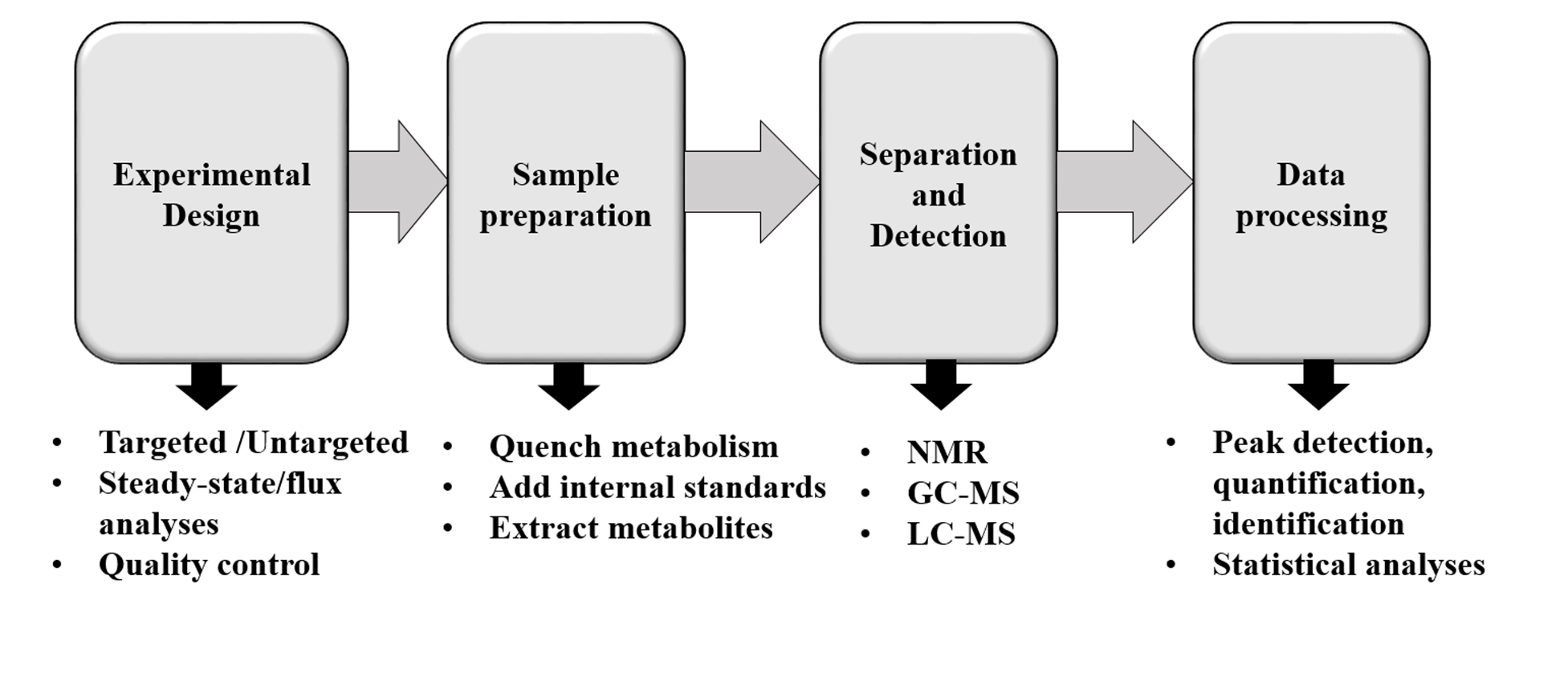


Figure S2

Supplement: Supplementary file 1 — Additional file 1: Fig. S1. Applications of metabolomics (modified from Gomez-Casati et al. 2013, metabolomics in plants and humans: applications in the prevention and diagnosis of diseases. BioMed Research International, 1–11). Fig. S2. Flow diagram of the metabolomics studies (modified from Sas et al. 2015, Metabolomics and diabetes: Analytical and computational approaches. American Diabetes Association, 64:718–732). [file 40643_2021_396_MOESM1_ESM.docx]
